# Supplementary material for: A nanofluidic knot factory based on compression of single DNA in nanochannels
Source: Nat Commun. 2018 Apr 17;9:1506. doi: 10.1038/s41467-018-03901-w (PMC5904144; doi:10.1038/s41467-018-03901-w)
Supplement: Supplementary file 1 — Supplementary Information [file 41467_2018_3901_MOESM1_ESM.pdf]

Supplementary Information:
A Nanofluidic Knot Factory based on Compression of Single DNA in Nanochannels

Amin et al.

Supplementary Figures

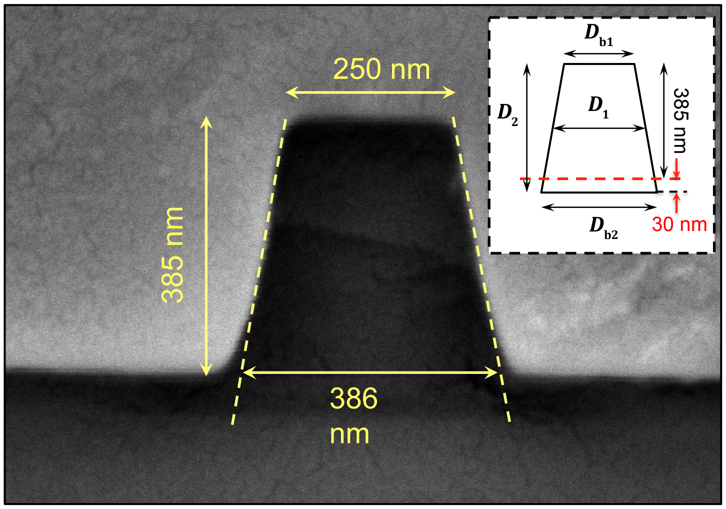

Supplementary Figure 1: Cross section of a nanochannel imaged using SEM; the cross section is a trapezoid with base dimensions D_{b1}, D_{b2} and height dimension D_2. The yellow dashed lines show the trapezoid edges. The inset shows a schematic of how the dimensions of the bonded chip are calculated. Adding the slit depth (measured with surface profilometer and shown in red) to the measured depth of the unbonded channel gives $D_2 \sim 415\,\mathrm{nm}$. The length $D_1 = 325\,\mathrm{nm}$ is the average of the base-lengths $D_{b1} = 250\,\mathrm{nm}$ and $D_{b2} = 386\,\mathrm{nm}$. The channel cross section is approximated as a rectangle with dimensions D_1 and D_2.

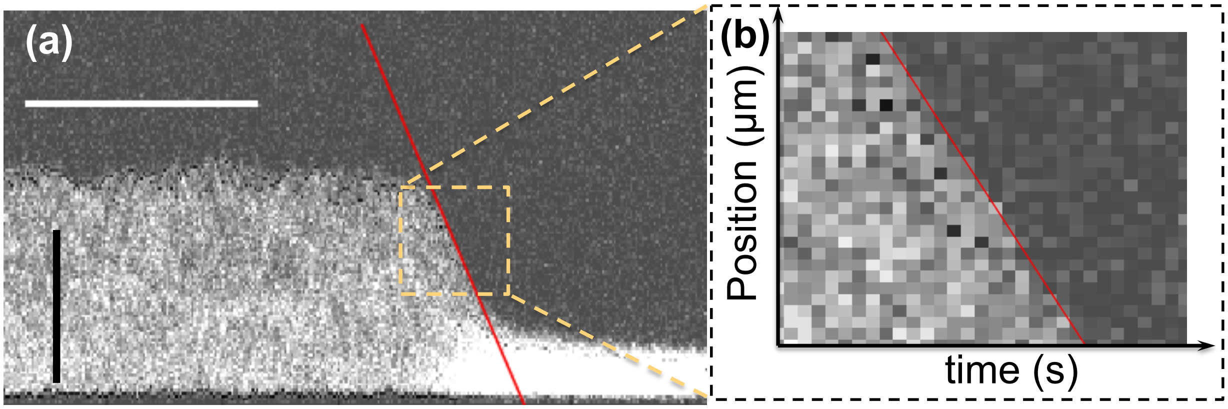

Supplementary Figure 2: Our approach for measuring speed of molecule free edge. The kymograph plot is created by reslicing the raw image to show intensity along the nanochannel versus time (horizontal axis shows time and the vertical axis position). The edge speed is calculated from a linear least-squares fit to the displacement of the molecule free end with time during transient compression. The horizontal and vertical scale bars are 0.5 s and 10 μm, respectively. (b) Magnified image of the kymograph; the slope of the line gives the speed.

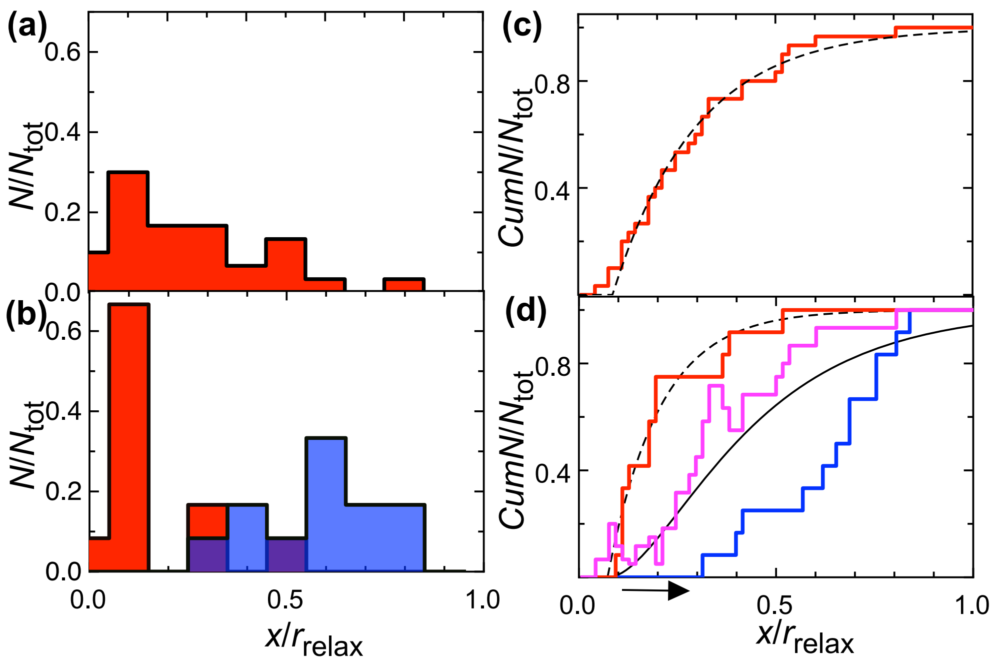

Supplementary Figure 3: Knot position histogram for one-knot states (a) and two-knot states (b) 2 sec after pressure release. Cumulative knot position histogram for one-knot states (c) and two-knot states (d) normalized to the total number of counts. The x-axis is normalized to the extension $r_{\rm relax}$ of the relaxing molecule measured at the time for which the knot-position was obtained ($x = 0$ gives barrier edge, $x = 1$ gives molecule free edge). The data used includes events with $R_{\rm b} \approx 0.11, 0.13, 0.17$. The ratio of the event averaged $r_{\rm relax}$ to the steady-state extension r is 2.9; $R_{\rm relax} \equiv r_{\rm relax}/r_{\rm o} = 0.5$. For the two-knot states, we separately histogram the position of the knot closest to the barrier ('lower knot', shown in red) and the knot farthest from the barrier ('upper knot', shown in blue). The dashed curve in (c) is a fit of the cumulative single-knot distribution to the cumulative distribution corresponding to an exponential probability distribution; the dashed curve in (d) is a fit of the cumulative lower-knot distribution to the cumulative distribution for an exponential probability distribution. The magenta bold curve in (d) is the estimated cumulative non-interacting upper knot distribution $C_{\rm U}$ formed directly using the single knot and lower knot measurements. The bold curve is the estimated cumulative non-interacting upper knot distribution $C_{\rm U}$ based on the exponential fit to the lower cumulative knot distribution. The arrow indicates that the upper knot distribution is shifted relative to the estimated $C_{\rm U}$ (bold black, bold magenta), indicating that, for at least at this particular time-point following relaxation, the lower- and upper-knots interact and they cannot pass.

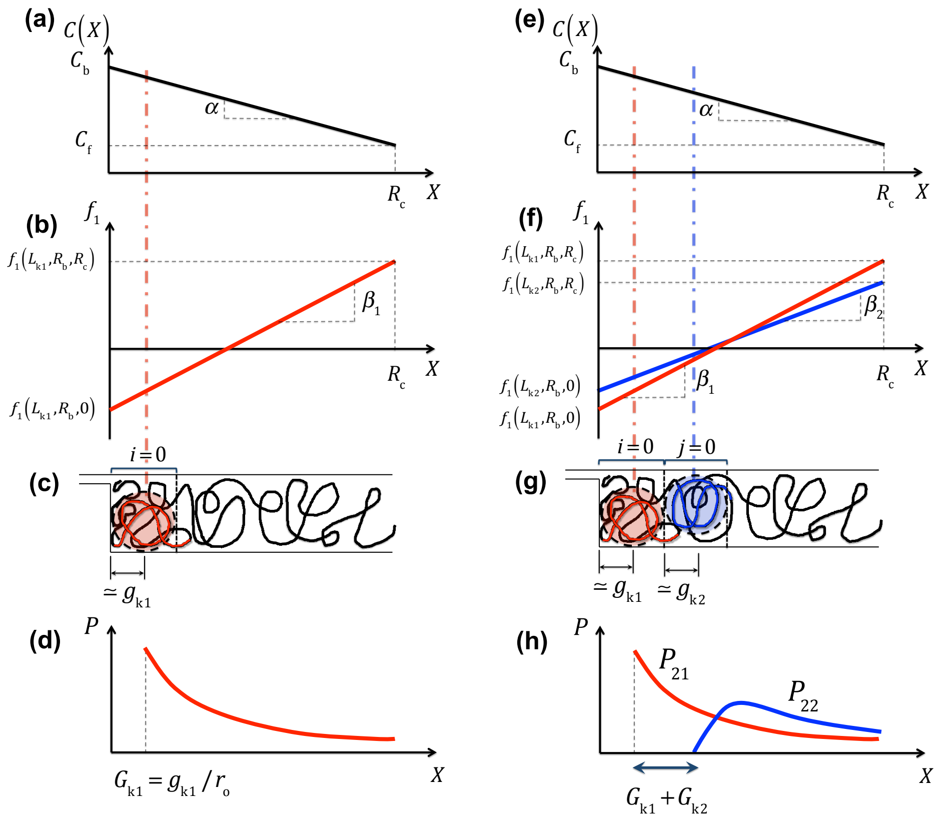

Supplementary Figure 4: (a, e) Schematic of concentration profile $C(X)$, (b, f) resulting free energy profile $f_1(L_\mathrm{k}, R_\mathrm{b}, X)$ and (d, h) resulting knot probability distributions for single (a-d) and two-knot (e-h) states. Note that in the two-knot state the two knots can experience different free energy profiles due to their varying size (e.g. $L_{\mathrm{k}1}$ does not necessarily equal $L_{\mathrm{k}2}$): this is indicated in (f) by drawing two free energy profiles with slopes β_1 and β_2. In addition, in the two-knot state the knots will have different probability distributions: P_{21} for the 'lower' knot closest to the barrier and P_{22} for the 'upper' knot farthest from the barrier (h). (c) Cartoon of single-knot on chain, displaced by radius of gyration $g_{\mathrm{k}1}$ from barrier edge. The red dashed line relates the knot position to the corresponding concentration and free energy: note that the steric interaction between the molecule and barrier edge leads to an increase $\beta G_{\mathrm{k}1}$ in the molecule free energy (recall that $G_{\mathrm{k}1} \equiv g_{\mathrm{k}1}/r_\mathrm{o}$). (g) Cartoon of two knots on chain. Knot 1 (red) is displaced by radius of gyration $g_{\mathrm{k}1}$ from barrier edge; knot 2 (blue), by assumption of single-file ordering, is displaced by $2g_{\mathrm{k}1} + g_{\mathrm{k}2}$ from barrier edge. The black dashed lines in (c, g) show the extent of each knot site on the compressed chain, used to calculate partition sums (See Supplementary Note 9). i and j denote site indices used in Supplementary Equations 50 and 52 for the first and second knots, respectively.

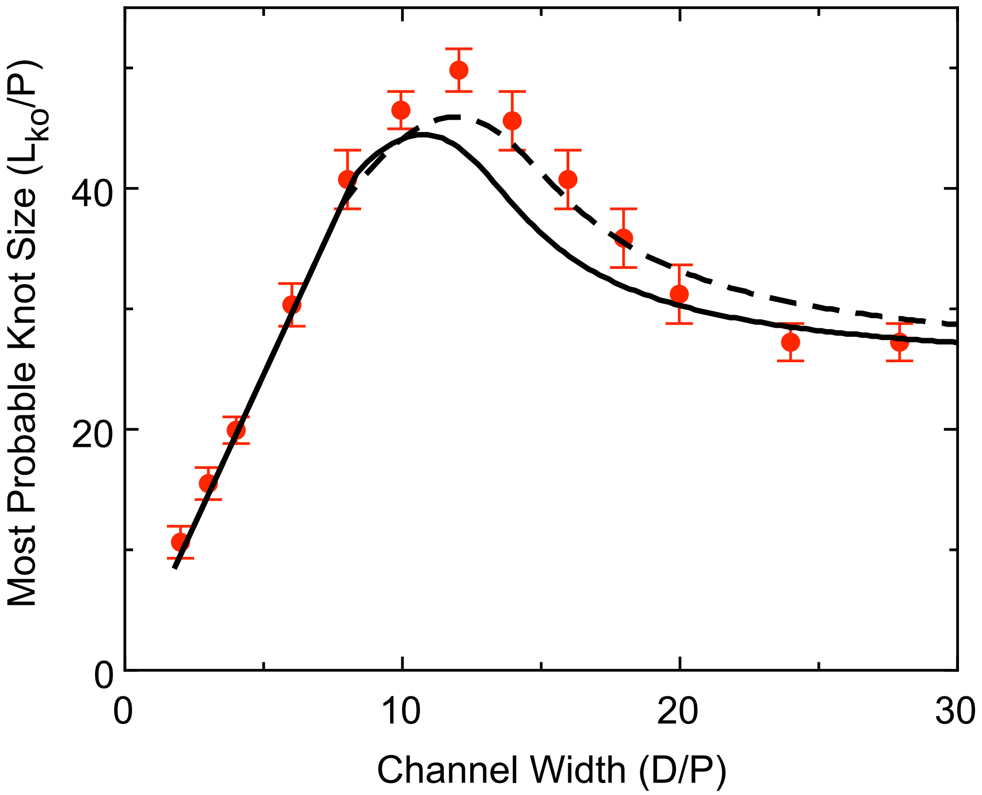

Supplementary Figure 5: Red circles show simulation results for most probable knot size from Dai *et al.* [1]. Bold curve shows prediction for most probable knot size found using Supplementary Equation 33 with $R = 1$ and $f_{\rm wuk}$ evaluated using Supplementary Equation 35 ($L_{\rm b}^{\rm o}$ determined via classic de Gennes theory). Dashed curve shows prediction for most probable knot size found using Supplementary Equation 33 with $R = 1$ and $f_{\rm wuk}$ evaluated using Supplementary Equation 35 ($L_{\rm b}^{\rm o}$ determined using the extended de Gennes theory). We have set $P = 0.4w$ to match the parameter values used in Dai *et al.*'s simulation. We have also increased the value of $A_{\rm wuk}$ by 1.5 to ensure that the free energy of the unknotted polymer includes the effect of the spring terms in Supplementary Equation 35 (in no-flow equilibrium, when $R = 1$, the quadratic spring term only has the effect of adjusting the scaling prefactor).

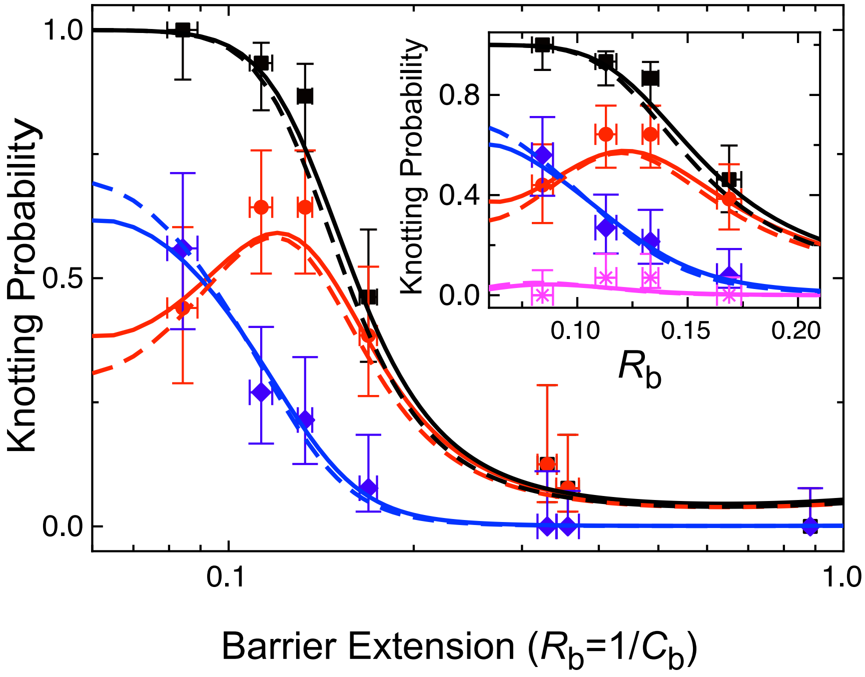

Supplementary Figure 6: The black squares give experimental measurements for total probability of forming an event with any number of knots. The red circles and blue diamonds give respectively measurements of one-knot and two-knot event probabilities. The magenta stars in the inset give probability measurements for three-knot events. The bold curves show the original three parameter fit yielding $A_{\rm b} = 1.43 \pm 0.05$, $A_{\rm wk} = 0.98 \pm 0.12$ and $A_{\rm h} = 1.12 \pm 0.07$. The dashed curves show a one-parameter fit fixing $A_{\rm wk} = 1$ and $A_{\rm h} = 1$ and fitting $A_{\rm b}$ (the approach gives $A_{\rm b} = 1.46 \pm 0.01$). The inset illustrates the predicted probability of knotting for a sample space including three-knot formation probability (linear-linear scale). Each data point is determined from the average result of ∼10-15 events. The vertical error bars on probability have been calculated using a Wilson-score interval with a one-sigma confidence interval [2]; the horizontal error bars represent the error on the mean for $R_{\rm b}$ measurements for the corresponding events.

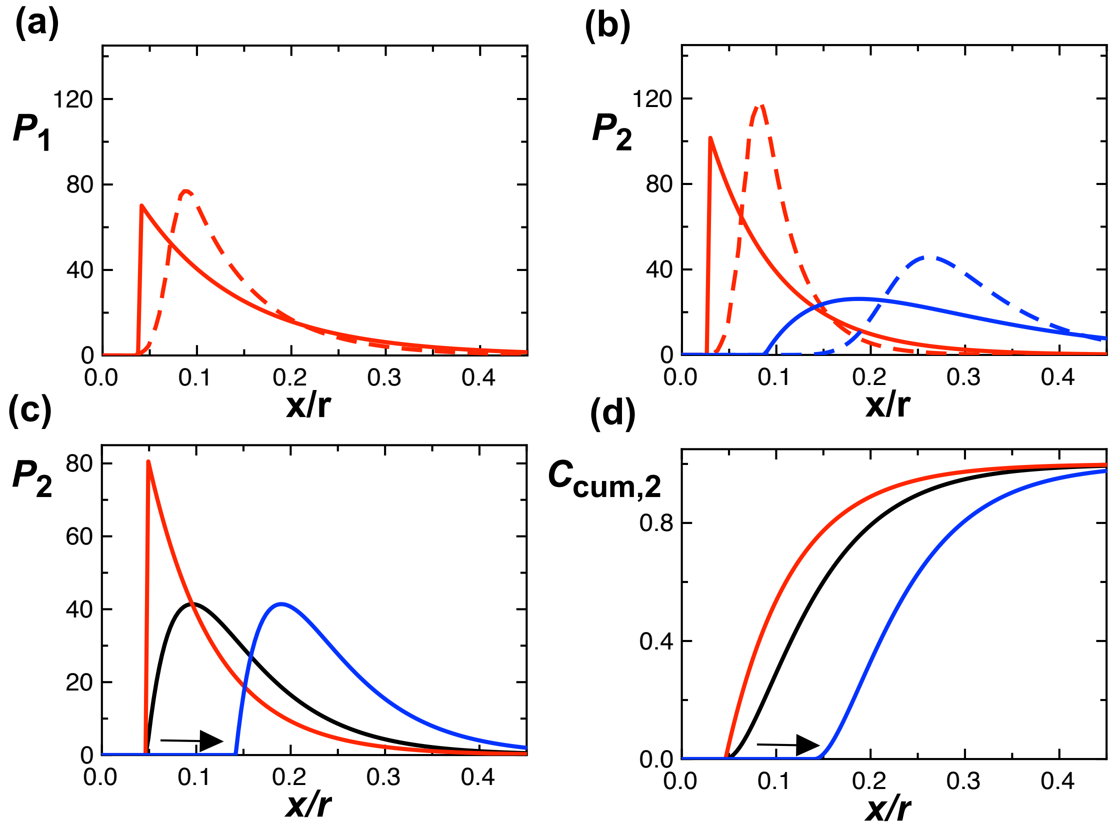

Supplementary Figure 7: (a) The predicted probability distribution of knot position in a one-knot state P_1 for a knot with the most probable knot size (bold) and averaging over all knots sizes (dashed). (b) The predicted probability distribution of knot position in a two-knot state for a lower knot with the most probable knot size (P_{21}, bold, red), averaging over all lower knot sizes ($P_{21}^{\rm av}$, dashed red), the most probable upper knot (P_{22}, bold, blue) and averaging over all upper knot sizes ($P_{22}^{\rm av}$, dashed, blue). (c) Example predicted probability distributions for knot position along profile for a two-knot state, showing lower knot distribution P_{21} (bold red), upper knot distribution P_{22} (bold blue) and the estimated non-interacting distribution $P_{\rm U}$ (bold black). (d) Example cumulative probability distributions for knot position along profile for a two-knot state, showing cumulative lower knot distribution C_{21} (bold red), cumulative upper knot distribution C_{22} (bold blue) and the estimated cumulative non-interacting distribution $C_{\rm U}$ (bold black). The arrows show the shift between the predicted non-interacting upper knot ($P_{\rm U}$, $C_{\rm U}$ black) and predicted interacting upper knot (P_{22}, C_{22} blue). Note that the lower knot distributions and non-interacting distributions in (c) and (d) (bold red, black curves) have been computed using the most probable size of knots in a one-knot state.

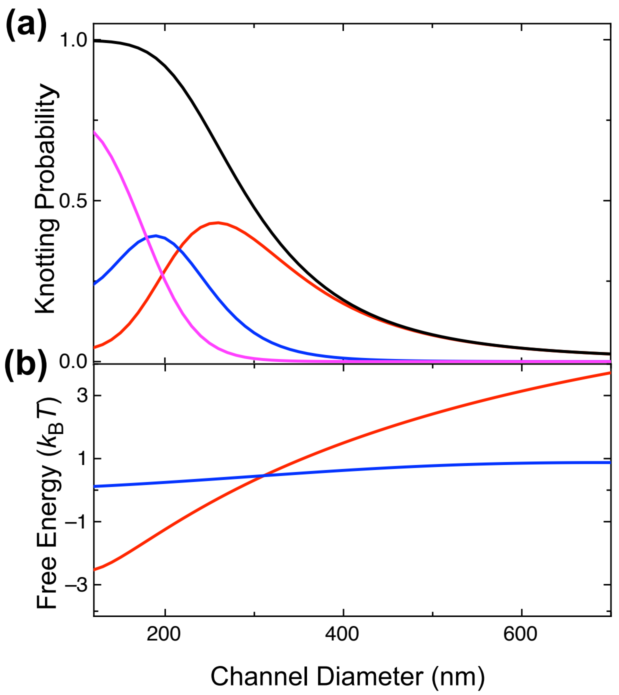

Supplementary Figure 8: (a) Predicted probability as a function of channel dimension of generating an event with one knot (red), two-knots (blue) and three-knot (magenta) on a profile with $R_b = 0.2$. Note that the large three-knot formation probability would predict that for channel widths below 200 nm, states with even more knots would be generated (although we do not include these explicitly in the calculation). The total probability of generating an event with knots is the black-curve. (b) The free energy of a single-knot state (red) and two-knot interaction free energy (blue) versus channel width. For simplicity, the channels have aspect ratio unity.

Supplementary Note 1

Nomenclature

α Slope of the steady-state ramp profile for a single chain compressed against the slit-barrier

$\beta(L_\mathrm{k}, R_\mathrm{b})$ Slope of the free energy profile for a molecule with edge extension R_b

Δp Pressure drop across the nanofluidic channel

ΔV Streaming potential

δ Offset in the channel dimension due to the presence of a knot

η Solution viscosity

γ A numerical factor relating the radius of gyration g_k to the offset in the channel dimension δ

$\hat{P}_i$ Maximum likelihood estimate for the probability of a knotting state with i knots

$\hat{P}_\mathrm{total}$ Maximum likelihood estimate of total knotting probability

λ Transition rate ratio k_{21}/k_{12}

Π Osmotic pressure

σ Width of the point spread function f_PSF

σ_c Buffer conductivity

σ_n Wilson score interval

σ_r Standard deviation in the extension at compressed state

$\xi_{||}$ Blob extent for a molecule in an extended de Gennes regime at no-flow equilibrium

ζ Friction factor per unit length

ζ_f Friction factor at molecule free edge

ζ_k Knot friction factor

$C(X)$ Normalized chain concentration at position X ($\equiv c(X)/c_o$)

$c(x,t)$ The average concentration profile over channel cross section at position x and time t. This quantity reduces to $c(x)$ in steady state.

C_b Normalized chain concentration at the barrier edge ($\equiv c_b/c_o$)

c_b Chain concentration at the barrier edge

$C_{cum,ij}(X_j)$ Cumulative distribution of the jth knot in i-knot event

$C_{cum}(x)$ The cumulative knot distribution for one-knot event

C_f Normalized chain concentration at the molecule free edge ($\equiv c_f/c_o$)

c_f Chain concentration at the molecule free edge

C_L Cumulative lower knot distribution (in two-knot events) assuming knots do not interact (i.e. non-interacting distribution)

c_o The concentration of the chain in the absence of flow ('no-flow' equilibrium concentration)

C_U Cumulative upper knot distribution (in two-knot events) assuming knots do not interact (i.e. non-interacting distribution)

D'_{eff} Channel effective diameter, taking into account the reduction in the channel size due to the presence of a knot

D_1 Horizontal dimension of the nanochannel cross section

D_2 Vertical dimension of the nanochannel cross section

D_{av} The geometrical average of nanochannel cross section

D_c Cooperative diffusion constant

D_{eff} Channel cross-section effective diameter

$f_i(L_{k1}, ..., L_{ki}, R_b, X_1, ..., X_i)$ Free energy of an i-knot state event with the knots on normalized positions on the chain X_1 to X_i

$F_m(L_{k1}, \cdots, L_{km}, R_b)$ Free energy for forming m knots of size $L_{k1}, \cdots, L_{km}$ along a compressed molecule with edge extension R_b

$f_b(L_k)$ Free energy cost of formation of a knot of contour L_k in bulk

$f_h(L_k, V, X)$ Free energy required to move knot away from the barrier ($X = 0$) against hydrodynamic flow

$f_{int}(X_2 - X_1)$ Free energy of interaction between two knots at normalized positions X_1 and X_2

f_{PSF} Point spread function

$F_{tot}(m, R_b)$ Free energy for forming m knots including all knot sizes for a compressed molecule with edge extension R_b

$f_{wk}(L_k, R)$ Free energy of confining a knot of contour L_k between the channel walls at a position on the molecule concentration profile with local extension R.

$f_{wuk}(L_k, R)$ Free energy of confining an unknotted chain of contour L_k between the channel walls at a position on the molecule concentration profile with local extension R.

$f_{ij}(X_j)$ Free energy of knot j at position X_j in the i-knot event

G_k Normalized knot gyration radius ($\equiv g_k/r_o$)

g_k Knot gyration radius

$I(x)$ Fluorescence intensity profile of molecule at position x on the chain

I_o Intensity level of the extended molecule at no-flow equilibrium

I_s Buffer ionic strength

J Total segmental current

J_c Convective segmental current along the channel

J_D Cooperative diffusion current along the channel

k_B Boltzmann constant

k_{ij} Transition rate from a state with i knots to a state with j knots

L_b Contour per blob in the no-flow equilibrium chain

L_c Channel length

L_k Knot contour length

n_k Maximum number of knots observed in an event

n_max The number of statistically independent sites at which a knot can form along the profile

P Persistence length

$P(i, R_\mathrm{b})$ The probability of finding a state with i knots along a compressed molecule with barrier edge extension R_b.

P^av Predicted non-interacting single-knot distribution averaged over all knot sizes

P_i Probability of formation of i knots

$P_i(L_{\mathrm{k}1}, ..., L_{\mathrm{k}i}, R_\mathrm{b}, X_1, ..., X_i)$ i-knot probability distribution for finding i knots at position $X_1, ..., X_i$ along a profile with edge extension R_b.

$P_\text{all knots}$ The probability of finding a state with any number (non-zero) of knots

$P_\mathrm{diff}(\delta x)$ Probability distribution for δx (the distance between two knots on a chain)

$P_\mathrm{L}(x)$ Probability of knot distribution for the lower knot (closer to the barrier) at position x in 2-knot event assuming knots do not interact (i.e. non-interacting distribution)

P_L^av Predicted non-interacting lower knot distribution averaged over all knot sizes

P_o High-salt persistence length

P_tot Total probability of knot formation

$P_{\mathrm{U}}(x)$ Probability of knot distribution for the upper knot (closer to the free edge) at position x in 2-knot event assuming knots do not interact (i.e. non-interacting distribution)

$P_{\mathrm{U}}^{\mathrm{av}}$ Predicted non-interacting upper knot distribution averaged over all knot sizes

$P_{ij}(X_j)$ The probability distribution for the position X_j of knot j in the i-knot space

r Molecule extension in compressed state

$R(X)$ Local extension at normalized position X on the chain ($\equiv 1/C(X)$)

R_{b} Local extension at the barrier ($\equiv 1/C_{\mathrm{b}} = 1/C(0)$)

R_{ch} Electrical resistance of the nanochannel

R_{c} Normalized chain extension ($= r/r_{\mathrm{o}}$)

r_{o} Molecule extension in the absence of flow ('no-flow' equilibrium extension)

r_{relax} Extension of the relaxing molecule measured 2 seconds after pressure release

S_{str} Streaming conductance

T Temperature

t_{w} Waiting time in compressed state

V Molecule free edge speed during transient compression: a measure of the buffer flow speed

w Effective width of the chain

X Normalized position on the chain ($\equiv x/r_{\mathrm{o}}$)

$x_{\mathrm{f}}(t)$ The position of the molecule free edge at time t

$Z(m, R_{\mathrm{b}})$ The partition function for a system with m knots

$z_m(L_{\mathrm{k1}}, \cdots, L_{\mathrm{k}m}, R_{\mathrm{b}})$ Partition sum for forming m knots of size $L_{\mathrm{k1}}, \cdots, L_{\mathrm{k}m}$ along a compressed molecule with edge extension R_{b}

$z_{\alpha/2}$ The critical value of the normal distribution for error level α

$Z_{\mathrm{tot}}(R_{\mathrm{b}})$ Total knot partition function for a compressed molecule with barrier extension R_{b}

Supplementary Note 2

Experimental Methods

In order to obtain the shape and dimensions of the nanochannel cross section, a chip is cut carefully at the center where the nanochannels are located: the sample is then diced half-way through using a wafer saw and then broken by hand about the saw cut to avoid destroying the nanochannels with the saw blade. A platinum layer of 4-nm thickness is sputtered on the cleaved sample. Upon imaging the chip using SEM, the cross-section shape is observed to be trapezoidal with base dimensions $D_{\mathrm{b1}} = 250\,\mathrm{nm}$ and $D_{\mathrm{b2}} = 386\,\mathrm{nm}$ (Supplementary Figure **1**). Adding the slit depth (measured with surface profilometer) to the measured depth of the unbonded channel gives $D_2 \sim 415\,\mathrm{nm}$ (Supplementary Figure **1**(inset)). Note that the trapezoidal area $A = D_2(D_{\mathrm{b1}} + D_{\mathrm{b2}})/2 \equiv D_2 D_1$ with the dimension $D_1 \equiv (D_{\mathrm{b1}} + D_{\mathrm{b2}})/2 = 325\,\mathrm{nm}$ (i.e. the average of the two base lengths). In other words, a rectangular cross-section of dimension D_1 and D_2, having the same area as the original channel, is the best rectangular approximation to our trapezoidal cross-section. The use of a non-unity aspect ratio requires that the channel width D must be replaced by the geometric average $D_{\mathrm{av}} = \sqrt{D_1 D_2}$ =367 nm [3].

We briefly discuss our approach for estimating the physical value of P and the effective width w for our buffer conditions. Our buffer has an estimated ionic strength of $8\,mM$, a value we obtain from solving the coupled chemical equilibria for Tris-HCl and BME ($\mathrm{pK_{tris}} = 8.1$ and $\mathrm{pK_{BME}} = 9.6$). The persistence length is estimated using Odijk-Skolnick-Fixman (OSF) theory [4, 5]:

$$P = P_{\mathrm{o}} + \frac{32\,\mathrm{mM}}{I_{\mathrm{s}}}\,\mathrm{nm} \tag{1}$$

with P_{o} the high salt persistence length ($P_{\mathrm{o}} = 51\,\mathrm{nm}$). We find $P = 55\,\mathrm{nm}$ at $I_{\mathrm{s}} = 8\dot{\mathrm{m}}\mathrm{M}$. The Stigter theory, which estimates the effective width of DNA based on the excluded-volume of charged rods, is used to evaluate the effective w [5, 6]. We find $w = 17\,\mathrm{nm}$. The high value of the effective width suppresses bulk knotting [7]; the only knots observed are those formed through compression.

Supplementary Note 3

Single-Chain Concentration Profiles: Data Analysis and Theoretical Models

A. Streaming Potential Estimation

In our experiments no external electric field is applied. While an electric field will be introduced by electrokinetic cross-coupling due to streaming current, these effects are expected to be very small. To estimate the order of magnitude of the streaming potential, we scale classic measurements of the streaming conductance performed in fused silica nanoslit devices [10]. The streaming conductance S_{str} gives the resulting current per unit of pressure applied across the channel, resulting from motion of ions in the Debye layers. Van der Heyden *et al.* [10] found that the streaming conductance S_{str} for $50\,\mu$m wide, 4.5 mm long fused silica nanoslits, with height between 279 nm and 563 nm, was in the range 20-30 pA bar^{-1}. To estimate the resulting streaming conductance for our nanochannel devices, we scale the nanoslit streaming conductance by the ratio of the nanochannel to nanoslit width ($325\,\mathrm{nm}/50\,\mu\mathrm{m}$) and multiply by 2 to take into account the Debye layer from the horizontal dimension of our channels (this is of course crude, but we are interested in an order of magnitude estimate). In our experiment, as there is no external load circuit, we expect that the streaming potential [11]

$$\Delta V = -S_{\mathrm{str}} \Delta p R_{\mathrm{ch}} \tag{2}$$

where Δp is the pressure drop across the nanofluidic channel and R_{ch} is the electrical resistance of the nanochannel ($R_{\mathrm{ch}} = L_{\mathrm{c}}/D_1 D_2 \sigma_{\mathrm{c}}$, with L_{c} channel length and σ_{c} the buffer conductivity). Using a buffer conductivity of $\sigma_{\mathrm{c}} = 1\,\mathrm{mS\,cm^{-1}}$, $L_{\mathrm{c}} = 200\mu\mathrm{m}$ and a pressure drop $\Delta p = 30\,\mathrm{mbar}$, corresponding to the highest flow achieved ($V = 30\mu\mathrm{m\,s^{-1}}$), we find that $\Delta V \sim 0.2\,\mathrm{mV}$. Alternatively, we can scale the direct streaming potential measurement in [11], taking into account our lower pressure and smaller channel length; this leads to $\Delta V \sim 0.3\,\mathrm{mV}$, which agrees on order of magnitude. The eletrokinetic mobility of DNA in our channels is around $2\,\mu\mathrm{m\,cm\,(sV)^{-1}}$. The electrokinetic DNA velocity resulting from dropping the streaming potential across the channel length is then $\sim 0.02\mu\mathrm{m\,s^{-1}}$, three orders of magnitude below the flow-speeds we observe.

B. Physics of Steady-State Compression

Here we justify in detail the analogy between hydrodynamic and piston-based compression and review relevant details from the past work [8, 9]. In our experiments, a nanochannel-extended chain is compressed against a barrier via steady hydrodynamic flow induced by creating a pressure drop across the channel. As the channel dimensions are much smaller than the total molecule extension, even at full compression, we adopt a coarse-grained one-dimensional approach, with the concentration $c(x)$ referring to an average of the segmental concentration over the channel cross-section. This scenario is highly analogous to compression of nanochannel-confined DNA via a translating piston-gasket moving with fixed sliding speed [8, 9] viewed in a reference frame co-moving with the piston. In the co-moving reference frame, the piston's sliding speed V is transformed into a uniform flow of speed V compressing the polymer against the piston-gasket (analogous to the slit-barrier). Now, if we could apply a perfectly uniform flow field in our channels, our hydrodynamic experiments would be identical to compression via the sliding piston. The difference is that, in the case of pressure-driven flow, the flow-profile is parabolic [12]. In this case, the relevant flow-speed is V_{av} arising from an average of the product of the parabolic Poiseuille flow profile across the channel and the molecule's transverse segmental distribution [13]. While the physical details that set the value of V_{av} are more complex, it behaves in our 1-D formalism analogous to the translation speed V. For convenience of notation, we choose to drop the subscript 'av' and write $V \equiv V_{\mathrm{av}}$. As in the case of the sliding piston, this flow creates a 1-D convective segmental current along the channel $J_{\mathrm{c}} = -Vc$ (the negative sign arises as in our coordinate system, with $x = 0$ at the barrier, flow drives segments to smaller x).

The convective flow is balanced by osmotic pressure gradients that drive segments from regions of high to low concentration. These osmotic pressure gradients lead to cooperative diffusion, characterized by the current:

$$J_{\mathrm{D}} = -D_{\mathrm{c}}(c)\frac{\partial c}{\partial x}. \tag{3}$$

The quantity $D_{\mathrm{c}}(c)$ is a *cooperative diffusion constant* and itself is a function of polymer concentration:

$$D_{\mathrm{c}} = \frac{cD_{\mathrm{av}}^2}{\zeta}\frac{\partial \Pi}{\partial c} \tag{4}$$

where ζ is a friction factor per unit length. D_{av} is the geometric average of the channel cross section. The osmotic pressure can be obtained from the Flory free energy via an argument detailed in [9]:

$$\Pi = \Pi_{\mathrm{o}} \left(-1/C + C^2\right) \tag{5}$$

The quantity $\Pi_{\mathrm{o}} \equiv Ak_{\mathrm{B}}T/(D_{\mathrm{av}}^2 \xi_{\|})$ with A a numerical constant ($A = 2.81$) and $\xi_{\|}$ the blob extent. The quantity $C \equiv c/c_{\mathrm{o}}$, where c_{o} is the concentration of the chain in the absence of flow. Experiment [9] and scaling considerations [8] suggest that $\zeta = \zeta_{\mathrm{o}} C$, which inserted in Supplementary Equation 4 and along with Supplementary Equation 5 lead to:

$$D_{\mathrm{c}} = \frac{2\Pi_{\mathrm{o}} D_{\mathrm{av}}^2}{\zeta_{\mathrm{o}}} C \left(1 + \frac{1}{2C^3}\right). \tag{6}$$

Note that the $1/2C^3$ term becomes negligible for $C > 2$ (true for the high degree of compression used in our knotting study) so that $D_{\mathrm{c}} \sim C$.

The dynamics of the compression process can be described using the nonlinear diffusion PDE formalism developed in [9]. We insert our expression for the total current $J = J_{\mathrm{D}} - cV$ (with J_{D} defined via Supplementary Equation 3) into a continuity equation expressing local conservation of polymer segments,

$$\frac{\partial c}{\partial t} + \frac{\partial J}{\partial x} = 0. \tag{7}$$

leading to a nonlinear partial differential equation for predicting the time-evolution of a chain's concentration profile $c(x, t)$:

$$\frac{\partial c}{\partial t} - \frac{\partial}{\partial x} \left(D_{\mathrm{c}} \frac{\partial c}{\partial x} + cV\right) = 0. \tag{8}$$

When a molecule is driven by a steady flow against a barrier, one of the molecule edges will be pinned at the barrier ($x = 0$). The second edge, which we call the "free edge" x_{f}, will slide towards the barrier. Following [9], we argue that the position of the free edge $x_{\mathrm{f}}(t)$ can be described via the equation:

$$\zeta_{\mathrm{f}} \frac{dx_{\mathrm{f}}}{dt} = \Pi(C(x_{\mathrm{f}})) D_{\mathrm{av}}^2 - \zeta_{\mathrm{f}} V. \tag{9}$$

In this picture, the free edge is driven by a combination of the flow-induced velocity and osmotic pressure exerted at the chain free edge. When $c = c_{\mathrm{o}}$ at the free edge so that $C(x_{\mathrm{f}}) = 1$, Supplementary Equation 5 predicts that $\Pi = 0$ and the free edge is driven at the speed V. The quantity ζ_{f} is the free-edge friction; we find that treating ζ_{f} as constant gives good description of experimental data for transient compression [9]. Initially, during the compression process, the free edge remains at concentration c_{o} and is observed to slide towards the barrier at constant velocity [9]. This edge velocity can then be accessed by finding the slope of a position versus time plot of the polymer free edge x_{f} during transient compression, providing a way to measure V experimentally that self-consistently incorporates unknown details regarding the transverse averaging (see Supplementary Note 3 (C) for detail on the velocity measurement).

While the free edge slides towards the barrier, contour builds up locally at the barrier, creating a transient shockwave. At a time on order of r_{o}/V, the free edge reaches the barrier and the profile evolves towards a steady-state ramp in the limit of long times. The steady-state profile can be described by setting $J = 0$:

$$D_{\mathrm{c}}(c)\frac{\partial c}{\partial x} = -Vc \tag{10}$$

Note that the ramp profile is determined by the functional form of $D_{\mathrm{c}}(c)$. If we let $D_{\mathrm{c}} \sim C$ the ramp is linear with a slope $\alpha \sim V$. In terms of the normalized position variable $X \equiv x/r_{\mathrm{o}}$, with the barrier edge at $X = 0$ corresponding to the most concentrated point on the chain with concentration $C_{\mathrm{b}} \equiv C(0) \geq C(x)$:

$$C(X) = C_{\mathrm{b}} - \alpha X. \tag{11}$$

The steady-state concentration at the free edge $c_{\mathrm{f}} \equiv c(r)$ is provided by the solution of Supplementary Equation 9 with $dx_{\mathrm{f}}/dt = 0$:

$$\Pi(C_{\mathrm{f}})D^2 = \zeta_{\mathrm{f}}V. \tag{12}$$

With $\Pi(C_{\mathrm{f}}) \sim C_{\mathrm{f}}^2$ (a good approximation for large C_{f}) and treating ζ_{f} as constant, we find that $C_{\mathrm{f}} \sim \sqrt{V}$.

The profile extension r can be found from conservation of contour (i.e. the integral of

$c(x)$ is constant [8]). We first define the normalized *chain extension* $R_\mathrm{c} \equiv r/r_\mathrm{o}$. Contour conservation, in terms of the dimensionless variables, can be expressed [9]:

$$\int_0^{R_\mathrm{c}} C(X)\, dX = 1. \tag{13}$$

The chain extension R_c can be determined as a function of V by combining Supplementary Equation 11 and Supplementary Equation 13 and working the integral. For high V we find $R_\mathrm{c} \sim 1/\sqrt{V}$ [8]. Using Supplementary Equation 11 we can relate the concentration at the barrier edge to the concentration at the free edge: $C_\mathrm{b} = C_\mathrm{f} + A_1 V R_\mathrm{c}$, so that $C_\mathrm{b} \sim \sqrt{V}$.

We choose to introduce a new variable $R(X) \equiv 1/C(X)$, which we call the *local extension*. The local extension measures how locally stretched ($R > 1$) or in our case how compressed ($R < 1$) the chain is relative to the no-flow equilibrium (where $R = 1$ at every point along the chain). We define the 'local barrier extension' $R_\mathrm{b} \equiv R(0)$. Note that $R_\mathrm{b} = 1/C_\mathrm{b} \sim 1/\sqrt{V}$. As $\alpha \sim V$, we can in turn write α as a function of R_b: $\alpha \sim 1/R_\mathrm{b}^2$. Holding all other physical variables constant (channel dimensions, effective width, persistence length) we need to select only one variable to completely parameterize the profile for varying degrees of compression (i.e. completely determine the slope and the edge concentration and thus the concentration at all positions via Supplementary Equation 11). While a natural choice is V or R_c, we choose the local barrier extension as this is the variable most closely linked to the knotting free energy (see Supplementary Note 7) but like R_c it transparently characterizes the chain's total degree of compression (e.g. small R_b corresponds to high compression, with $R_\mathrm{b} \leq R(X)$ for all X). In fact, our theory predicts R_b is proportional to R_c, which we will show in the final paragraph of this section.

Figure 2(e-h) in the manuscript shows experimental results for the steady-state profiles. We show results for all compression events without binning. Figure 2(e) in the manuscript confirms that the data is consistent with a linear relation between α and V; Fig. 2(f) in the manuscript confirms that our results are consistent with $C_\mathrm{b} \sim \sqrt{V}$ and Fig. 2(h) in the manuscript confirms $\alpha \sim 1/R_\mathrm{b}^2$. For definiteness, let

$$\alpha = A_1 V \tag{14}$$

$$C_\mathrm{b} = A_2 \sqrt{V} \tag{15}$$

and

$$\alpha = A_3/R_{\rm b}^2. \tag{16}$$

From least squares-fitting we find $A_1 = 3 \pm 0.1$, $A_2 = 2.52 \pm 0.04$ and $A_3 = 0.464 \pm 0.004$.

Lastly, we will prove that $R_{\rm b}$ is proportional to $R_{\rm c}$ and use the values of A_1 and A_2 to estimate the proportionality factor. Integrating Supplementary Equation 13 with the concentration profile determined by Supplementary Equation 11 and using Supplementary Equation 14 and Supplementary Equation 15 we find that:

$$1 = C_{\rm b} R_{\rm c} - \frac{1}{2}\alpha R_{\rm c}^2 \Rightarrow R_{\rm b} = 1/C_{\rm b} = \left[\frac{A_1}{A_2}\frac{1}{A_2 - \sqrt{A_2^2 - 2A_1}}\right] R_{\rm c} \equiv A_4 R_{\rm c} \tag{17}$$

Supplementary Equation 17 gives the proportionality factor $A_4 = 0.62 \pm 0.05$. We can also estimate the proportionality factor directly from experiment, from the slope of measurements of $R_{\rm b}$ plotted versus $R_{\rm c}$. We find $A_4 = 0.64 \pm 0.01$, which agrees with the estimate from Supplementary Equation 17.

C. Molecule Free-Edge Speed Measurement

We use the motion of the free molecule edge, unconstrained during the transient compression phase, as a measure of the buffer flow-speed. Note that during the first phase of the compression process, the free edge remains at concentration $c_{\rm o}$ and is expected to move towards the barrier at constant speed V [9] (See Supplementary Equation 5 and Supplementary Equation 9). In agreement with this prediction, we observe that the measured free-edge position vs. time is linear (See Supplementary Figure **2**). The free edge speed V is then extracted from the slope of a linear fit to the free edge position (via *lsqcurvefit* in Matlab).

D. Intensity Profile Fitting Functions

A nanochannel-extended molecule in its no-flow equilibrium conformation has a uniform concentration profile. Upon hydrodynamic compression against the slit barriers, the DNA molecules will evolve towards a steady-state concentration profile that has a ramp-like char-

acter (see Fig. 1(g-j) in the manuscript). We find that this profile is well-described by a linear ramp form (Eq. 1 and Fig. 1(k) in the manuscript). An experimental difficulty is that we cannot resolve the exact theoretical concentration profile $c(x)$ as our optics are diffraction limited. Instead, we image broadened profiles formed by convolving the theoretical profile with a point-spread function $f_{\mathrm{PSF}}(x)$ [8, 9]. A second difficulty is that we measure fluorescence intensity $I(x)$, not concentration. To deduce the broadened experimental profiles from a given predicted profile $c(x)$, we use:

$$I(x) = C \int_{-\infty}^{\infty} c(y) f_{\mathrm{PSF}}(x-y)\, dy. \tag{18}$$

For simplicity, we choose a Gaussian form for $f_{\mathrm{PSF}}(x)$:

$$f_{\mathrm{PSF}}(x) = \frac{1}{\sqrt{2\pi\sigma^2}} \exp\left(-\frac{x^2}{2\sigma^2}\right). \tag{19}$$

The PSF width σ is estimated by fitting to the profiles. The constant C in Supplementary Equation 18 is a calibration factor representing the conversion between concentration and intensity level (depending on factors such as stain ratio, illumination power-levels, stain quantum efficiency and the stain extinction coefficient). By using $I/I_{\mathrm{o}} = c/c_{\mathrm{o}}$, we remove the calibration factor from consideration.

We use two functional forms for the underlying concentration profiles $c(x)$. The uniform-profile shape, appropriate for an equilibrium no-flow profile, is defined by:

$$c_{\mathrm{flat}}(x) = \begin{cases} c_{\mathrm{o}} & \text{if } x > x_1 \text{ and } x < x_2 \\ 0 & \text{otherwise} \end{cases} \tag{20}$$

The extension is simply the difference between the profile-end points: $r = x_2 - x_1$. The ramp profile shape is defined by:

$$c_{\mathrm{ramp}}(x) = \begin{cases} c_{\mathrm{b}} - A_{\mathrm{r}} x & \text{if } x > x_1 \text{ and } x < x_2 \\ 0 & \text{otherwise} \end{cases} \tag{21}$$

The quantity A_{r} is the profile ramp-rate. By applying Supplementary Equation 18 and Supplementary Equation 19 to Supplementary Equation 20 and Supplementary Equa-

tion 21, we can deduce analytic forms (expressed in terms of error-functions and Gaussians) for the broadened profiles. All profile parameters, such as extension, ramp-rate, maximum/minimum concentration and σ can be obtained by fitting experimental profiles to these broadened functional forms (see Fig. 1(k) in the manuscript for an example fit to experiment of a broadened ramp along with an estimate of the underlying ramp-profile).

E. Waiting time

Waiting time is defined as the duration for which a molecule is held in a compressed state. Each molecule is compressed to a certain minimum extension for a certain waiting time and then relaxed. To create a consistent measurement of the waiting time, we first find the average extension of the molecule at equilibrium at compressed state r and the standard deviation σ_r in the extension. Starting from the first frame of the movie, once the extension of the molecule reaches a value within the interval $[r - \sigma_\mathrm{r}, r + \sigma_\mathrm{r}]$, we set that frame as $t = 0$. The point of pressure release determines the waiting time $t = t_\mathrm{w}$.

Supplementary Note 4

Knot Formation Rate Equations

The knot formation kinetics can be described by a set of coupled rate equations. Defining P_i as the probability of formation of i knots and k_{ij} as the transition rate from a state with i knots to a state with j knots, we have

$$P_0 \underset{k_{10}}{\overset{k_{01}}{\rightleftharpoons}} P_1 \underset{k_{21}}{\overset{k_{12}}{\rightleftharpoons}} P_2. \tag{22}$$

The explicit system of rate equations following from Supplementary Equation 22 is

$$\frac{dP_0}{dt_\mathrm{w}} = -P_0 k_{01} + P_1 k_{10} \tag{23a}$$

$$\frac{dP_1}{dt_\mathrm{w}} = P_0 k_{01} + P_2 k_{21} - P_1(k_{10} + k_{12}) \tag{23b}$$

$$\frac{dP_2}{dt_\mathrm{w}} = P_1 k_{12} - P_2 k_{21} \tag{23c}$$

Note that as $t_\mathrm{w} \to \infty$ the system reaches equilibrium and the time derivatives vanish giving rise to the three equations:

$$P_0(\infty)k_{01} = P_1(\infty)k_{10} \tag{24a}$$

$$P_0(\infty)k_{01} + P_2(\infty)k_{21} = P_1(\infty)(k_{10} + k_{12}) \tag{24b}$$

$$P_1(\infty)k_{12} = P_2(\infty)k_{21} \tag{24c}$$

Our experimental waiting time data was taken at high compression for which $P_0(\infty) \approx 0$. Thus the backwards rate $k_{10} \approx 0$. In addition, we have $P_1(\infty)/P_2(\infty) = k_{21}/k_{12}$. Letting

$\lambda = k_{21}/k_{12}$ the system can then be solved exactly,

$$P_1(t_\mathrm{w}) = \frac{\left[\left(e^{-k_{01}t_\mathrm{w}} - e^{-(1+\lambda)k_{12}t_\mathrm{w}} + \lambda\left(e^{-k_{01}t_\mathrm{w}} - 1\right)\right)k_{01} + \left(1 - e^{-k_{01}t_\mathrm{w}}\right)\lambda(1+\lambda)k_{12}\right]}{(1+\lambda)(-k_{01} + k_{12}(1+\lambda))} \tag{25}$$

$$P_2(t) = \frac{[k_{01}(-1 + e^{-k_{12}(1+\lambda)t_\mathrm{w}}) + k_{12}(1+\lambda)(1 - e^{-k_{01}t_\mathrm{w}})]}{(1+\lambda)(-k_{01} + k_{12}(1+\lambda))} \tag{26}$$

The total probability of knot formation is $P_\mathrm{tot} = 1 - P_0(t_\mathrm{w}) = 1 - e^{-k_{01}t_\mathrm{w}}$. The three parameters k_{01}, k_{12} and λ are found by least-square fitting of the model to the experimental data (Fig. 3(b) in the manuscript), also using *lsqcurvefit*.

Supplementary Note 5

Knot-Spatial Distribution

The spatial distribution of knots can be accessed at a short-time following pressure release. Supplementary Figure **3** (which has identical data to Fig. 6 in the manuscript) shows the histogrammed position of knots for one-knot (Supplementary Figure **3**(a,c)) and two-knot events (Supplementary Figure **3**(b,d)) 2 sec after pressure release. For the two-knot events, the position of the knot closest to the slit-barrier ('lower-knot') and the knot farthest from the slit-barrier ('upper knot') are separately histogrammed. The figure shows knot histograms (Supplementary Figure **3**(a,b)) yielding an estimate of the knot spatial probability distribution $P_{ij}(X_j)$ (i gives knot-state, i.e. $i = 1$ for one knot state, $i = 2$ for two knot; j indexes the knot number in the state). The cumulative knot histogram $C_{\mathrm{cum},ij}(X_j)$ (Supplementary Figure **3**(c,d)) is simply the total number of knots observed at positions X' with $X' < X_j$. The cumulative distribution has the advantage that it is insensitive to binning and is preferred for use in data interpretation. To demonstrate rigorously that interactions exist, let us assume that the knots do not interact so that their spatial probability distributions are statistically independent. Moreover, assume that the positions of knots 1 and 2 follow the same probability density function $P(x)$. Note that, even in the absence of interactions (i.e. the knots can freely pass and cannot be distinguished based on their ordering along the profile), the lower and upper knot distributions will have different functional forms. The upper non-interacting knot distribution $P_{\mathrm{U}}(x)$ is given by

$$P_{\mathrm{U}}(x) = P(x_1 = x)P(x_2 \leq x) + P(x_2 = x)P(x_1 \leq x). \tag{27}$$

The first term is the probability that position x is the highest spatial position of a knot on the profile and that $x_1 > x_2$. This term is the product of the probability $P(x_1 = x) = P(x)$ that knot 1 is at position x and $P(x_2 \leq x)$ the probability that knot 2 is at a position $x_2 \leq x$ (e.g. given by $\int_0^x P(x_2)\,dx_2$). The second term is the symmetric contribution assuming $x_2 > x_1$. By similar reasoning, the lower knot non-interacting knot distribution $P_{\mathrm{L}}(x)$:

$$P_{\mathrm{L}}(x) = P(x_1 = x)P(x_2 \geq x) + P(x_2 = x)P(x_1 \geq x) \tag{28}$$

with the probability that knot 2 is at position $x_2 \geq x$ given by $P(x_2 \geq x) = \int_x^\infty P(x_2)\,dx_2$.

Note that, using $P(x_1 \leq x) + P(x_1 \geq x) = 1$, Supplementary Equations 27, 28 imply:

$$P_{\rm L}(x) + P_{\rm U}(x) = 2P(x) \tag{29}$$

To make this argument concrete, let $P(x)$ have an exponential form, as expected for the one-knot distributions in steady-state:

$$P(x) \sim \exp(-\beta x). \tag{30}$$

Evaluating Supplementary Equation 27 and Supplementary Equation 28 with $P(x)$ given by Supplementary Equation 30, we find that

$$P_{\rm L}(x) \sim \exp(-2\beta x) \tag{31}$$

and

$$P_{\rm U}(x) \sim (1 - \exp(-\beta x)) \exp(-\beta x) \tag{32}$$

Thus, shape-differences between the upper and lower distributions do not necessarily indicate that the knots interact, we expect $P_{\rm U}$ and $P_{\rm L}$ to have different forms even if no interactions are present. Note that the cumulative lower and upper knot distributions $C_{\rm L}$ and $C_{\rm U}$ can be formed from $P_{\rm L}$ and $P_{\rm U}$ via $C_{\rm cum}(x) = \int_0^x P(x')\,dx'$.

How then can we tell if interactions are present between the knots? The key is to look for differences between the predicted *non-interacting* upper knot distribution $P_{\rm U}$, and the measured upper knot distribution. If the knots do not interact by assumption, then the underlying distribution P must be identical for both knots and is given by the one knot distribution P_1; $P_{\rm L}$ can be obtained from the measured lower knot distribution and $P_{\rm U}$ is then necessarily determined by Supplementary Equation 29. If the obtained distribution $P_{\rm U}$ fails to describe the experimental upper knot distribution, then we can say interactions are present.

To test for knot interactions using our empirical knot distributions, we obtain $P_{\rm U}$ using the non-interacting assumption and compare it to the measured upper knot distribution (Supplementary Figure **3**(d)). The comparison is best made using the cumulative distributions ($C_{\rm U}$–magenta bold curve, blue curve–measured cumulative upper knot distribution).

Alternatively, we can estimate P_{U} by noting that an exponential shape well describes the lower-knot distribution (dashed curve Supplementary Figure **3**(d)), consistent with Supplementary Equation 31. The extracted parameter β (see Supplementary Equation 31) can then be used to estimate the distribution P_{U} via Supplementary Equation 32 (bold-line, Supplementary Figure **3**(d)). We see that the measured distribution is shifted to higher X relative to P_{U} obtained with both approaches (again, the comparison is best made with the cumulative distributions). This proves interactions exist at 2 sec following pressure release and these interactions are strong enough to prevent knot crossing, consistent with our hypothesis regarding knot-interactions in steady-state and single-file ordering of knots.

Furthermore, note that our basic interaction test–which is independent of model assumptions–is not altered by averaging over knot sizes (we expect a distribution of knot sizes to be present during the experiment). Let the predicted non-interacting lower, upper and single-knot distributions averaged over all knot sizes be denoted by $P_{\mathrm{L}}^{\mathrm{av}}$, $P_{\mathrm{U}}^{\mathrm{av}}$ and P^{av}. Then, averaging both sides of Supplementary Equation 29, we find that $P_{\mathrm{L}}^{\mathrm{av}}(x) + P_{\mathrm{U}}^{\mathrm{av}}(x) = 2P^{\mathrm{av}}(x)$. As P^{av} and $P_{\mathrm{U}}^{\mathrm{av}}$ will be obtained from the experimental knot data automatically (the experimental data is necessarily averaged over all knot sizes!), our argument in fact yields $P_{\mathrm{U}}^{\mathrm{av}}$, so the comparison is already being made with the averaged distributions.

Supplementary Note 6

Expanded Discussion of Knot Formation Free Energy

In this section, we expand the discussion of the knot free energy model developed in the manuscript. Our objective is to compute the probability of forming i knots on an extended chain in terms of the edge extension R_{b}. Our model is based very closely on the approach of Dai *et al.* [1]. In this section, we discuss in detail the types of free energy associated with a nanochannel-confined knot. The free energy of a single knot will be expressed as a function of knot contour length L_{k}, position along the profile X and the edge extension R_{b}. Note that the edge extension serves to completely parameterize the concentration profile $C(X)$ obtained at a given flow speed V. In the following sections, we will discuss the procedure for spatial averaging over X, including interaction between knots for multi-knot states and forming partition functions that sum over L_{k} and can be compared directly to experimental knotting probabilities.

Three types of free energy are associated with a knot of size L_{k} on a nanochannel-confined chain [1]. The first contribution is the free energy *cost* of forming the knot on the chain in bulk $f_{\mathrm{b}}(L_{\mathrm{k}})$. The second contribution is the free energy *cost* of confining the knot between the channel walls $f_{\mathrm{wk}}(L_{\mathrm{k}}, R)$ ('wall-knot'). The third contribution is the free energy *saved* as contour stored in the knot no longer contributes the confinement free energy $f_{\mathrm{wuk}}(L_{\mathrm{k}}, R)$ ('wall-unknot') associated with an unknotted section of polymer of size L_{k}. Note that the bulk contribution depends only on the knot size L_{k}. The wall-knot and wall-unknot contributions depend on L_{k} *and* the local extension $R(X) = 1/C(X)$. The local extension is in turn a function of position X and the edge extension R_{b} through the profile (Supplementary Equation 11). Note that we see no evidence that the existence of knots alters the profile-shape. In addition, the knot will have an extra free energy $f_{\mathrm{h}}(L_{\mathrm{k}}, V, X)$ due to the flow V, which exerts a constant viscous drag force pulling knots to lower X and thus raising their free energy when they are displaced from the slit-barrier. Combining the terms, one knot of size L_{k} at position X on a profile with edge extension R_{b} has a total free energy:

$$\begin{aligned} f_1(L_{\mathrm{k}}, R_{\mathrm{b}}, X) = {} & A_{\mathrm{b}} f_{\mathrm{b}}(L_{\mathrm{k}}) + A_{\mathrm{wk}} f_{\mathrm{wk}}(L_{\mathrm{k}}, R(X, R_{\mathrm{b}})) \\ & - f_{\mathrm{wuk}}(L_{\mathrm{k}}, R(X, R_{\mathrm{b}})) + A_{\mathrm{h}} f_{\mathrm{h}}(L_{\mathrm{k}}, R_{\mathrm{b}}, X). \end{aligned} \tag{33}$$

We choose to explicitly include the dimensionless scaling prefactors $A_{\rm b}$, $A_{\rm wk}$ and $A_{\rm h}$ associated with the bulk, wall-knot and hydrodynamic terms: these will be determined by fitting to experimental knotting probability data. Following Dai *et al.*, the existing theory will be used to explicitly calculate the scaling prefactor $A_{\rm wuk}$ as this term is the well-known confinement free energy of a nanochannel-confined polymer. Also, note that the free energy of the unknotted chain represents the base-line free energy for this theory: explicitly f_1 gives the excess (or decrease) in free energy arising from the presence of one knot on the chain. By definition we have $f_0 = 0$. In order to simplify the notation, all free energy terms will be expressed in units of $k_{\rm B}T$. Note also that we express f_1 and $f_{\rm h}$ as a function purely of X, $L_{\rm k}$ and $R_{\rm b}$: the flow speed V can be eliminated using Supplementary Equation 15 and Supplementary Equation 16.

Dai *et al.* have used a combination of scaling arguments and simulation to deduce the detailed forms for the free energy contributions in Supplementary Equation 33. The term $f_{\rm b}$ gives an R-independent contribution that can be obtained from a balance of bending and confinement free energy [14, 15]:

$$f_{\rm b}(L_{\rm k}) = k_1 (L_{\rm k}/P)^{-1} + k_2 L_{\rm k} \left(L_{\rm k} - pw\right)^{-2/3} P^{-1/3}, \tag{34}$$

where P and w are the persistence length and effective width of the chain, respectively. The quantities k_1, k_2 and p are numerical constants determined from simulations of equilibrium trefoil knots ($k_1 = 17.06$, $k_2 = 1.86$ and $p = 16$ [15]).

The contribution $f_{\rm wuk}$ can be obtained from the Flory free energy for a confined chain [16]:

$$f_{\rm wuk}(L_{\rm k}, R) = A_{\rm wuk} \frac{L_{\rm k}}{L_{\rm b}} \left(R^2/2 + 1/R\right) \tag{35}$$

This equation is derived for a uniformly extended chain (e.g. R = constant), but we can interpret Supplementary Equation 35 in a local sense for a chain with a non-uniform concentration profile as giving the free energy of confinement associated with a small contour $L_{\rm k}$ extracted from position X along the profile with local extension $R(X)$. The quantity $L_{\rm b}$ is the contour per blob in the equilibrium chain, which we obtain from extended de Gennes theory (the appropriate confinement regime for channel in our size-range): $L_{\rm b} = \left(2P/w^2\right)^{1/3} D_{\rm eff}^{4/3}$ with P the persistence length and w the effective width (we estimate $P = 55\,{\rm nm}$ and $w = 17\,{\rm nm}$ (Supplementary Note 2). The quantity $D_{\rm eff} = \sqrt{(D_1 - w)(D_2 - w)}$ is an effec-

tive diameter taking into account the non-unity aspect ratio of the channel and wall-DNA interactions (estimating the electrostatic depletion near the channel walls by w [3]). Note that, in the classic de Gennes regime, $L_{\mathrm{b}} = D_{\mathrm{eff}}^{5/3}/(2Pw)^{1/3}$. The quantity A_{wuk} is a numerical constant ($A_{\mathrm{wuk}} = 2.81$ [16]). Note that Supplementary Equation 35 does differ subtly from the unknot free energy used in [1]; Dai *et al.*, focusing mostly on channels of larger size than those used in our study, use a classic de Gennes assumption, while an extended de Gennes assumption is more appropriate for the channel size used here. Lastly, as the knot formation probability is only appreciable below $R = 0.5$, we argue that the R^2 term in Supplementary Equation 35 is negligible for our experiments.

Dai *et al.* observe in their simulations that the knots can be viewed as spherical regions with radius of gyration g_{k}. The relation between g_{k} and L_{k} can be determined from Monte Carlo simulation of knotted semiflexible and self-avoiding chains. Dai *et al.* find that the relation $g_{\mathrm{k}} = 0.1L_{\mathrm{k}}$ describes their simulation results well (see in particular the supplementary material to [1]). The free energy cost f_{wk} arises from two sources: the knot, acting like a hard sphere, interacts via excluded volume with the rest of the chain; the knot also experiences greater confinement due to its physical size, effectively reducing the channel width. In order to quantify these effects, we adopt the 'knot-chain' approach of Dai *et al.*. In the knot-chain approach, the confinement free energy of the knot is given by the confinement free energy of one bead on an effective chain made up of bead-units of radius g_{k}. For the case of an uncompressed chain, where $R = 1$, Dai *et al.* argue that

$$f_{\mathrm{wk}} = L_{\mathrm{k}} \frac{g_{\mathrm{k}}^{2/3}}{D_{\mathrm{eff}}'^{5/3}} \tag{36}$$

The quantity $D'_{\mathrm{eff}} = \sqrt{(D_1 - \delta)(D_2 - \delta)}$ takes into account the increase in effective confinement felt by the knot, arising from the fact that the cross-section of the channel occupied by the chain must be reduced by the finite size of the knot, introducing an offset $\delta = \gamma g_{\mathrm{k}}$ with the value $\gamma = 1$ describing their simulations well. We have generalized Supplementary Equation 36 from the result given by Dai *et al.* to account for our channel's non-unity aspect ratio. Supplementary Equation 36 exactly reproduces Eq. 6 in [1] in the limit that $D_1 = D_2$.

Dai *et al.*'s original result (Supplementary Equation 36) requires modification when the chain is compressed ($R < 1$). The confinement free energy f_{wk} arises in part from excluded volume interactions between the knot and the rest of the chain. This free energy cost in-

creases as the chain is compressed, due to the decreasing available chain volume, introducing a dependence on R (again, f_{wk} is interpreted locally as the free energy associated with a knot of size L_{k} at position X with local extension $R(X)$). For $R > 0.5$, reference [16] suggests

$$f_{\mathrm{wk}}(L_{\mathrm{k}}, R) = L_{\mathrm{k}} \frac{g_{\mathrm{k}}^{2/3}}{D_{\mathrm{eff}}'^{5/3}} \left(R^2/2 + 1/R\right) \tag{37}$$

Note that the compression factor has the same form as Supplementary Equation 35. For higher compression, [16] suggests the physics enters a 'semidilute' regime where the dependence changes to $\sim 1/R^{5/4}$ [16]:

$$f_{\mathrm{wk}}(L_{\mathrm{k}}, R) = L_{\mathrm{k}} \frac{g_{\mathrm{k}}^{2/3}}{D_{\mathrm{eff}}'^{5/3}} \frac{1}{R^{5/4}} \tag{38}$$

We will use Supplementary Equation 38 as in our data the knot formation events were mostly in the high compression regime. Note that this semidilute regime does not apply to f_{wuk}, the confinement free energy of the unknotted polymer, which should follow extended de Gennes statistics (e.g due to the large P/w ratio and consequent anisotropy of the polymer segments) and enter a mean-field regime at higher compression for which the $\sim 1/R$ scaling is correct.

Assuming that hydrodynamic interactions are likely present over the knot-size, which is on order of the channel diameter, we estimate the friction factor of the confined knot as that of a sphere of radius g_{k}. The knot friction factor is then $\zeta_{\mathrm{k}} = 6\pi\eta g_{\mathrm{k}}$ with η viscosity ($\sim 1\,\mathrm{mPs}$) leading to a drag force $6\pi\eta g_{\mathrm{k}} V$ so that the hydrodynamic term f_{h} (in units of $k_{\mathrm{B}}T$) is:

$$f_{\mathrm{h}}(L_{\mathrm{k}}, V, X) = 6\pi\eta g_{\mathrm{k}} V r_{\mathrm{o}} X / k_{\mathrm{B}}T \tag{39}$$

where η is the solution viscosity. Note that we can choose to write the flow speed V as a function of the edge extension using Supplementary Equation 15 and Supplementary Equation 16, so that the single knot free energy f_1 can be completely expressed as a function of L_{k}, X and R_{b}.

Supplementary Note 7

Spatial Variation of Knotting Free Energy

The free energy of confined knots depends on their position along the profile (see Supplementary Figure **4**). This dependence arises from $R(X) = 1/C(X)$ and the explicit X-dependence of the hydrodynamic term $f_{\mathrm{h}}(L_{\mathrm{k}}, V, X)$. While Supplementary Equations 11, 35 and 39 indicate that f_{wuk} and f_{h} depend linearly on X, Supplementary Equation 38 implies that $f_{\mathrm{wk}} \sim R^{-5/4}$, introducing a slight nonlinearity. We treat the nonlinearity by Taylor expanding f_{wk} for small X:

$$f_{\mathrm{wk}}(L_{\mathrm{k}}, R(X, R_{\mathrm{b}})) = L_{\mathrm{k}} g_{\mathrm{k}}^{2/3} D_{\mathrm{eff}}^{-5/3} R^{-5/4} \approx L_{\mathrm{k}} g_{\mathrm{k}}^{2/3} D_{\mathrm{eff}}^{-5/3} \left[R_{\mathrm{b}}^{-5/4} - \frac{5}{4} \alpha R_{\mathrm{b}}^{-1/4} X \right] \tag{40}$$

This approximation is justified by the exponential dependence of one-knot formation probability on f_1 (Supplementary Equation 33) through Boltzmann statistics, ensuring that the probability is only appreciable for small X where the Taylor expansion is valid. Combining Supplementary Equations 11, 33, 34, 35, 38, 39 and 40 leads to:

$$f_1(L_{\mathrm{k}}, R_{\mathrm{b}}, X) = f_1(L_{\mathrm{k}}, R_{\mathrm{b}}, 0) + \beta(L_{\mathrm{k}}, R_{\mathrm{b}}) X \tag{41}$$

with

$$f_1(L_{\mathrm{k}}, R_{\mathrm{b}}, 0) = A_{\mathrm{b}} f_{\mathrm{b}}(L_{\mathrm{k}}) + A_{\mathrm{wk}} f_{\mathrm{wk}}(L_{\mathrm{k}}, R_{\mathrm{b}}) - f_{\mathrm{wuk}}(L_{\mathrm{k}}, R_{\mathrm{b}}) \tag{42}$$

and the slope of the free energy profile $\beta(L_{\mathrm{k}}, R_{\mathrm{b}})$ given by:

$$\beta(L_{\mathrm{k}}, R_{\mathrm{b}}) = \alpha \left(f_{\mathrm{wuk}}(L_{\mathrm{k}}, 1) - \frac{5 A_{\mathrm{wk}}}{4} f_{\mathrm{wk}}(L_{\mathrm{k}}, 1) R_{\mathrm{b}}^{-1/4} \right) + A_{\mathrm{h}} f_{\mathrm{h}}(L_{\mathrm{k}}, 1) \tag{43}$$

Both $\alpha \sim V \sim 1/R_{\mathrm{b}}^2$ and $f_{\mathrm{h}} \sim V \sim 1/R_{\mathrm{b}}^2$ (see Supplementary Equations 14, 15 and 16) so that $\beta \cong 1/R_{\mathrm{b}}^2$ (the scaling is only approximate due to the factor of $R_{\mathrm{b}}^{-1/4}$ from the Taylor expansion of f_{wk}).

Supplementary Note 8

Probability Distributions for Knot Position for Single and Two-Knot States

Supplementary Equation 41 implies that single knots have an exponential probability distribution in position-space: $P_1(L_\mathrm{k}, R_\mathrm{b}, X) \sim \exp\left(-\beta(L_\mathrm{k}, R_\mathrm{b})X\right)$ (see Supplementary Figure **4**(d)). While the knots are likely to be found near $X = 0$ and $f_1(L_\mathrm{k}, R_\mathrm{b}, 0)$ provides a good estimate of the free energy of single-knot states, the free energy will be slightly higher due to thermal excitation of knots to higher X (described by $P_1(L_\mathrm{k}, R_\mathrm{b}, X)$) and the finite size of the knots (the knots will 'bump' into the slit barrier at $x = g_\mathrm{k}$ forcing them to have higher free energy, Supplementary Figure **4**(b, c)).

In order to gain a quantitative description of states with multiple knots, we must include knot interactions. Including interactions prevents the formation of states with a very large number of knots even if the single knot formation probability is high. Knot-knot interactions, for example, could arise through the excluded volume of one knot restricting the configuration space of the other knots ('knot-knot' excluded volume); this effect would scale as $k_\mathrm{B}T g_\mathrm{k}^3/rD_1D_2$. Yet, the volume of a single knot is very small relative to the volume occupied by the chain: with $g_\mathrm{k} \sim 100\,\mathrm{nm}$ we find $g_\mathrm{k}^3/rD_1D_2 \sim 10^{-2}$. The knot-knot interactions must be at least on order of a fraction of $k_\mathrm{B}T$ as they have an appreciable effect after only two knots are present (see Fig. 4(b) in the manuscript). We believe the interactions must thus have a more subtle physical origin.

We argue that the knots do interact via hard-wall repulsion over their diameter $2g_\mathrm{k}$, as in a typical excluded-volume scenario, but that the effect of this interaction is greatly magnified by the channel confinement. In particular, we argue that inside the channel the knots satisfy a *no-crossing* condition: knots cannot pass in either contour or position space, leading to single-file diffusion of knots in the linear potential $f_1(L_\mathrm{k}, R_\mathrm{b}, X)$. While it is believed that molecular knots can pass through each other in bulk by diffusion along the chain contour [17], this mechanism requires an overall swelling of knot-size. In order to sufficiently lower the free energy barrier, so one knot can diffuse through the second knot, one knot must swell to almost its original size plus that of the second knot. Inside a channel, this mechanism cannot be operative if the knots have a diameter on order of the channel width. Thus, it is reasonable to argue that the knots behave like interacting hard spheres subject to a no-crossing condition via excluded-volume considerations alone.

With single-file ordering assumed, the knot interaction energy arises from a very simple mechanism: multiple knots stack in the linear potential, pushing knots to higher X and thus higher position in the free energy ramp (see Supplementary Figure **4**(f, g)). We can make this idea rigorous using the approach of [18]. Firstly, we define the notion of normalized knot gyration radius $G_{\mathrm{k}} \equiv g_{\mathrm{k}}/r_{\mathrm{o}}$. Say two knots of contour L_{k1} and L_{k2} with corresponding normalized gyration radii G_{k1} and G_{k2} are at normalized positions X_1 and X_2. In our linear approximation, we can say the knots have free energies $f_{11}(X_1) = f_{11}^{\mathrm{o}} + \beta_1 X_1$ and $f_{21}(X_2) = f_{21}^{\mathrm{o}} + \beta_2 X_2$ with $f_{11}^{\mathrm{o}} \equiv f_1(L_{\mathrm{k1}}, R_{\mathrm{b}}, 0)$, $f_{21}^{\mathrm{o}} \equiv f_1(L_2, R_{\mathrm{b}}, 0)$, $\beta_1 \equiv \beta(L_{\mathrm{k1}}, R_{\mathrm{b}})$ and $\beta_2 \equiv \beta(L_{\mathrm{k2}}, R_{\mathrm{b}})$. In addition, the knots interact via a potential $f_{\mathrm{int}}(X_2 - X_1)$ which has the hard-core form:

$$f_{\mathrm{int}}(X_2 - X_1) = \begin{cases} 0 & \text{if } X_2 - X_1 > G_{\mathrm{k1}} + G_{\mathrm{k2}} \\ \infty & \text{if } X_2 - X_1 \leq G_{\mathrm{k1}} + G_{\mathrm{k2}} \end{cases} \tag{44}$$

The total free energy of the two interacting knots is then:

$$f_2(L_{\mathrm{k1}}, L_{\mathrm{k2}}, R_{\mathrm{b}}, X_1, X_2) = f_{11}(X_1) + f_{21}(X_2) + f_{\mathrm{int}}(X_2 - X_1). \tag{45}$$

Defining $\delta X \equiv X_2 - X_1$, we can rewrite Supplementary Equation 45:

$$f_2 = \underbrace{[f_{11}^{\mathrm{o}} + f_{21}^{\mathrm{o}} + (\beta_1 + \beta_2)X_1]}_{=f'(X_1)} + \underbrace{[\beta_2 \delta X + f_{\mathrm{int}}(\delta X)]}_{=f''(\delta X)} \tag{46}$$

In other words, when rewritten in terms of X_1 and δX, the free energy can be expressed as the sum of the contributions $f'(X_1)$ and $f''(\delta X)$ so that the probability distributions of the variables X_1 and δX are independent. Using the notation $P_{21}(X_1)$ to indicate the probability distribution of knot 1 in the two-knot state, and using Supplementary Equation 44, we can write:

$$P_{21}(X_1) = \begin{cases} 0 & \text{if } X_1 \leq G_{\mathrm{k1}} \\ N_{21} \exp\left(-(\beta_1 + \beta_2)X_1\right) & \text{if } X_1 > G_{\mathrm{k1}} \end{cases} \tag{47}$$

The probability distribution $P_{\mathrm{diff}}(\delta X)$ for δX is:

$$P_{\mathrm{diff}}(\delta X) = \begin{cases} 0 & \text{if } \delta X \leq (G_{\mathrm{k1}} + G_{\mathrm{k2}}) \\ N_{\mathrm{diff}} \exp\left(-\beta_2 \delta X\right) & \text{if } \delta X > (G_{\mathrm{k1}} + G_{\mathrm{k2}}) \end{cases} \tag{48}$$

with N_{diff} and N_{21} normalization constants.

Lastly, we can deduce the probability distribution of knot 2 in the two-knot state P_{22}. Recalling $X_2 = \delta X + X_1$, we observe that X_2 is expressed as the sum of the independent random variables δX and X_1 described respectively by the probability distributions $P_{\text{diff}}(\delta X)$ (Supplementary Equation 48) and $P_{21}(X_1)$ (Supplementary Equation 47). Thus, $P_{22}(X_2)$ is determined by the *convolution* of $P_{\text{diff}}(\delta X)$ and $P_{21}(X_1)$. Using Supplementary Equation 47 and Supplementary Equation 48,

$$\begin{aligned} P_{22}(X_2) &= \int P_{\text{diff}}(Y) P_{21}(X_2 - Y)\, dY \\ &= N_{21} N_{\text{diff}} \int_{G_{\text{k1}}+G_{\text{k2}}}^{X_2 - G_{\text{k1}}} e^{-\beta_2 Y} e^{-(X_2 - Y)(\beta_1+\beta_2)}\, dY \\ &= N_{22} e^{-(\beta_1+\beta_2)X_2} \left(-e^{\left(G_{\text{k1}}+G_{\text{k2}}\right)\beta_1} + e^{\left(-G_{\text{k1}}+X_2\right)\beta_1} \right) \end{aligned} \tag{49}$$

with N_{22} a normalization factor. The lower integration limit is determined by the hard-wall cut-off in Supplementary Equation 48. The upper limit is the maximum value of δX for fixed X_2 (δX is maximized at fixed X_2 when $X_1 = G_{\text{k1}}$). Note that P_{21} and P_{22} do not reduce to their respective one-knot distributions $P_1(x) \sim \exp(-\beta_1 X)$ when $G_{\text{k1}} = G_{\text{k2}} = 0$ as the integration limits still enforce $\delta X > 0$ so that $X_2 \geq X_1$ (i.e. the model still assumes that the knots are not allowed to pass). The lower distribution P_{21} and upper distribution P_{22} are shown in Supplementary Figure **4**(h). Note that P_{22} is shifted from the origin.

Supplementary Note 9

Partition Functions for Knot Formation

In order to compare with our knot probability measurements we must compute partition functions that count the number of ways multiple knots can form on a compressed DNA profile: these partition functions involve summations over knot positions along the profile *and* knot size. We will start by considering summation over possible knot formation positions. Let the partition sum $z_1(L_{\mathrm{k}}, R_{\mathrm{b}})$ count the total number of ways a single prime knot of size L_{k}, assumed to be of trefoil topology, can form on a profile characterized by R_{b}. The number of statistically independent sites at which a knot can form along the profile is estimated by $n_{\max} = r/2g_{\mathrm{k}} = R/2G_{\mathrm{k}}$, each site i displaced by $\Delta X_i = 2G_{\mathrm{k}}(i+1/2)$ from $X = 0$. Note that i runs from 0 to $n_{\max} - 1$, with the first site at $i = 0$ displaced by a factor of G_{k} from the barrier due to the knot's finite spatial extent (see Supplementary Figure **4**(c)). Each site is weighted by a Boltzmann factor $\exp\left(-f_1(L_{\mathrm{k}}, R_{\mathrm{b}}, \Delta X_i)\right)$. Supplementary Equation 41 then leads to a single knot partition function:

$$z_1(L_{\mathrm{k}}, R_{\mathrm{b}}) = \sum_{i=0}^{n_{\max}-1} e^{-f_1(L_{\mathrm{k1}}, R_{\mathrm{b}}, \Delta X_i)} = e^{-f_1(L_{\mathrm{k1}}, R_{\mathrm{b}}, 0)} \sum_{i=0}^{n_{\max}-1} e^{-2\beta G_{\mathrm{k}}\left(i+\frac{1}{2}\right)} \tag{50}$$

where $\beta \equiv \beta(L_{\mathrm{k}}, R_{\mathrm{b}})$. As the probability of finding a knot away from the barrier is suppressed exponentially, it is a good approximation to set the sum limit to infinity, in which case we can sum Supplementary Equation 50 geometrically to find:

$$z_1(L_{\mathrm{k}}, R_{\mathrm{b}}) = \frac{e^{-f_1(L_{\mathrm{k1}}, R_{\mathrm{b}}, 0)+\beta G_{\mathrm{k}}}}{e^{2\beta G_{\mathrm{k}}} - 1}. \tag{51}$$

In the limit that the slope of the free energy profile is very steep, i.e. $\beta(L_{\mathrm{k}}, R_{\mathrm{b}})$ is large, Supplementary Equation 51 leads to a single knot free energy $F_1(L_{\mathrm{k}}, R_{\mathrm{b}}) = -\log\left(z_1(L_{\mathrm{k}}, R_{\mathrm{b}})\right) = f_1(L_{\mathrm{k1}}, R_{\mathrm{b}}, 0) + \beta(L_{\mathrm{k}}, R_{\mathrm{b}})G_{\mathrm{k}}$. This limit corresponds to a "ground state" configuration where knot formation at the minimum free energy position abutting the barrier dominates so that the knot free energy is simply the free energy at $X = 0$ ($f_1(L_{\mathrm{k1}}, R_{\mathrm{b}}, 0)$) plus the free energy increase arising from the knot's displacement by a factor of G_{k} "up the ramp" from $X = 0$ ($\beta(L_{\mathrm{k}}, R_{\mathrm{b}})G_{\mathrm{k}}$, see Supplementary Figure **4**(c)).

The partition function $z_2(L_{\mathrm{k1}}, L_{\mathrm{k2}}, R_{\mathrm{b}})$ counts the total number of ways two trefoil knots can form on a profile characterized by R_{b}. When multiple knots form we must construct

partition sums that preserve the linear ordering of the knots along the profile. Let $\Delta X_i = 2G_{\mathrm{k1}}(i+1/2)$ represent the displacement of the knot closest to the barrier from $X=0$ (with i running from 0 to $n_{\max}-1$ as before). The second knot must have a displacement $\Delta X_j \geq \Delta X_i + G_{\mathrm{k1}} + G_{\mathrm{k2}}$ leading to $\Delta X_j = 2G_{\mathrm{k1}}(i+1) + 2G_{\mathrm{k2}}(j+1/2)$. Each state (i,j) will then be weighted by a Boltzmann factor $\exp\left[-f_1(L_{\mathrm{k1}}, R_{\mathrm{b}}, \Delta X_i) - f_1(L_{\mathrm{k2}}, R_{\mathrm{b}}, \Delta X_j)\right]$. Making the approximation that the summation limits can be extended to infinity, Supplementary Equation 41 gives:

$$z_2(L_{\mathrm{k1}}, L_{\mathrm{k2}}, R_{\mathrm{b}}) = e^{-[f_1(L_{\mathrm{k1}},R_{\mathrm{b}},0)+f_1(L_{\mathrm{k2}},R_{\mathrm{b}},0)]} \sum_{i=0}^{\infty}\sum_{j=0}^{\infty} e^{-\left[2\beta_1 G_{\mathrm{k1}}(i+1/2)+2\beta_2\left(G_{\mathrm{k1}}(i+1)+G_{\mathrm{k2}}(j+1/2)\right)\right]} \tag{52}$$

with $\beta_1 \equiv \beta(L_{\mathrm{k1}}, R_{\mathrm{b}})$ and $\beta_2 \equiv \beta(L_{\mathrm{k2}}, R_{\mathrm{b}})$. The same procedure gives the partition function of a three-knot state:

$$\begin{aligned} z_3(L_{\mathrm{k1}}, L_{\mathrm{k2}}, L_{\mathrm{k3}}, R_{\mathrm{b}}) = {} & e^{-\left[f_1(L_{\mathrm{k1}},R_{\mathrm{b}},0)+f_1(L_{\mathrm{k2}},R_{\mathrm{b}},0)+f_1(L_{\mathrm{k3}},R_{\mathrm{b}},0)\right]} \\ & \times \sum_{i=0}^{\infty}\sum_{j=0}^{\infty}\sum_{l=0}^{\infty}\Bigg(e^{-2\beta_1 G_{\mathrm{k1}}(i+1/2)} \\ & \times e^{-2\beta_2\left(G_{\mathrm{k1}}(i+1)+G_{\mathrm{k2}}(j+1/2)\right)} \\ & \times e^{-2\beta_3\left(G_{\mathrm{k1}}(i+1)+G_{\mathrm{k2}}(j+1)+G_{\mathrm{k3}}(l+1/2)\right)}\Bigg). \end{aligned} \tag{53}$$

The partition sums in the Supplementary Equations 52, 53 can be evaluated by geometric summation:

$$z_2(L_{\mathrm{k1}}, L_{\mathrm{k2}}, R_{\mathrm{b}}) = \frac{e^{-f_1(L_{\mathrm{k1}},R_{\mathrm{b}},0)-f_1(L_{\mathrm{k2}},R_{\mathrm{b}},0)+G_{\mathrm{k1}}\beta_1+G_{\mathrm{k2}}\beta_2}}{\left(e^{2G_{\mathrm{k2}}\beta_2}-1\right)\left(e^{2G_{\mathrm{k1}}(\beta_1+\beta_2)}-1\right)} \tag{54}$$

and

$$z_3(L_{\mathrm{k1}}, L_{\mathrm{k2}}, L_{\mathrm{k3}}, R_{\mathrm{b}}) = \frac{e^{-f_1(L_{\mathrm{k1}},R_{\mathrm{b}},0)-f_1(L_{\mathrm{k2}},R_{\mathrm{b}},0)-f_1(L_{\mathrm{k3}},R_{\mathrm{b}},0)+G_{\mathrm{k1}}\beta_1+G_{\mathrm{k2}}\beta_2+G_{\mathrm{k3}}\beta_3}}{\left(e^{2G_{\mathrm{k3}}\beta_3}-1\right)\left(e^{2G_{\mathrm{k2}}(\beta_2+\beta_3)}-1\right)\left(e^{2G_{\mathrm{k1}}(\beta_1+\beta_2+\beta_3)}-1\right)} \tag{55}$$

In the limit that the slopes are very high, we have the following free energy for the two-knot and three-knot states:

$$F_2(L_{\mathrm{k1}}, L_{\mathrm{k2}}, R_{\mathrm{b}}) = -\log(z_2) = f_1(L_{\mathrm{k1}}, R_{\mathrm{b}}, 0) + f_1(L_{\mathrm{k2}}, R_{\mathrm{b}}, 0) + G_{\mathrm{k1}}\beta_1 + \left(G_{\mathrm{k2}} + 2G_{\mathrm{k1}}\right)\beta_2 \tag{56}$$

and

$$F_3(L_{k1}, L_{k2}, L_{k3}, R_b) = -\log(z_3) = f_1(L_{k1}, R_b, 0) + f_1(L_{k2}, R_b, 0) + f_3(L_{k3}, R_b, 0) + G_{k1}\beta_1 + (2G_{k1} + G_{k2})\,\beta_2 + (2G_{k1} + 2G_{k2} + G_{k3})\,\beta_3 \tag{57}$$

Supplementary Equations 56 and 57 correspond to the free energy of "ground states" where the knots are stacked in single file, with no gaps, directly abutting the barrier (Supplementary Figure **4**(g) gives the ground state for a configuration with two-knots).

As our knot-formation measurements include all knot sizes, the final step is to compute partition sums formed from integrating over the space of all knot sizes. We define $Z(m, R_b)$, the partition function for a system with m knots:

$$Z(m, R_b) = (2P)^{-m} \int z_m(L_{k1}, \cdots, L_{km}, R_b) dL_{k1} \cdots dL_{km} \tag{58}$$

We normalize to the Kuhn length to ensure that the partition function is dimensionless. Note that the total free energy associated with a knotting event with m knots (i.e. the free energy including all knot-sizes) is given by

$$F_{tot}(m, R_b) = -\log Z(m, R_b) \tag{59}$$

The partition function for finding zero knots $Z(0, R_b) = 1$. We can define a total knot partition function:

$$Z_{tot}(R_b) = \sum_{i=1}^{n_k} Z(i, R_b) \tag{60}$$

where n_k is the maximum number of knots observed in an event. The probability of finding a state with m knots is then:

$$P(m, R_b) = Z(m, R_b)/Z_{tot}(R_b) \tag{61}$$

The probability of finding a state with *any* number of knots (i.e. a state with at least one knot) is:

$$P_{\text{all knots}}(R_b) = \frac{Z_{tot}(R_b)}{1 + Z_{tot}(R_b)}. \tag{62}$$

This model can be readily solved via direct numerical integration of Supplementary Equa-

tion 58 with $z_m(L_{\mathrm{k}1}, \cdots, L_{\mathrm{k}m}, R_{\mathrm{b}})$ determined by Supplementary Equations 33, 34, 35, 38, and 39 (determine knot free energy contributions), Supplementary Equations 41-43 (determine knot free energy profile) and lastly Supplementary Equation 51, Supplementary Equation 54 and Supplementary Equation 55 (knot formation partitions functions for knots of fixed size). In practice, this integration is performed in Matlab by simply evaluating the partition function z_m for a finely sampled range of knot-sizes and summing. The prefactors A_{b}, A_{wk} and A_{h} are then determined from least-squares fitting to experimental data on one- and two-knot-formation probability.

Supplementary Note 10

Knot Statistics in the Low Compression Limit

The knot statistics are Poissonian in the limit that the compression is low and β is small. This can be easily shown analytically if we assume that the knots have equal size. The general form of the partition function for m trefoil knots on a profile characterized by R_{b} can be summarized by:

$$z_m(L_{\mathrm{k}1}, \cdots L_{\mathrm{k}m}, R_{\mathrm{b}}) = \frac{e^{-\sum_{i=1}^{m} f_1(L_{\mathrm{k}i}, R_{\mathrm{b}}, 0)} e^{\sum_{i=1}^{m} G_{\mathrm{k}i}\beta_i}}{\prod_{i=0}^{m-1}\left(e^{2G_{\mathrm{k},m-i}\sum_{j=0}^{i}\beta_{m-j}} - 1\right)} \tag{63}$$

Assuming that the knots have equal size $G_{\mathrm{k}1} = G_{\mathrm{k}2} = \ldots = G_{\mathrm{k}m} \equiv G_{\mathrm{k}}$, $\beta_1 = \beta_2 = \ldots = \beta_m \equiv \beta$ and $f_1(L_{\mathrm{k}1}, R_{\mathrm{b}}, 0) = f_1(L_{\mathrm{k}2}, R_{\mathrm{b}}, 0) = \ldots = f_1(L_{\mathrm{k}m}, R_{\mathrm{b}}, 0) \equiv f_1$, so we can rewrite Supplementary Equation 63 as,

$$z_m = \frac{e^{-mf_1 + mG_{\mathrm{k}}\beta}}{\prod_{i=1}^{m}\left(e^{2iG_{\mathrm{k}}\beta} - 1\right)}. \tag{64}$$

Supplementary Equation 64 gives the partition sum for the formation of m identical knots on a compressed DNA molecule. In the limit of low compression, hence small free energy slope β, we can approximate Supplementary Equation 64 using Taylor expansion,

$$z_m \approx \frac{e^{-mf_1 + mG_{\mathrm{k}}\beta}}{(2G_{\mathrm{k}}\beta)^m m!} = \frac{1}{m!}\left[\frac{e^{-f_1 + G_{\mathrm{k}}\beta}}{2G_{\mathrm{k}}\beta}\right]^m = \frac{1}{m!} z_1(L_{\mathrm{k}}, R_{\mathrm{b}})^m \tag{65}$$

The probability of forming m knots is then

$$P(m) = z_m / \sum_{j=0}^{\infty} z_j = \frac{1}{m!} z_1^m e^{-z_1} \tag{66}$$

which has the form of a Poisson distribution. In the inset to Fig. 5 in the manuscript we show numerically that partition functions that include all knot sizes also show Poisson behavior for low compression.

Supplementary Note 11

Comparison to Experiment

Our knot formation theory agrees well with the experimental measurements of knotting probability (Fig. 4 in the manuscript). In particular, we capture the increasing knot formation probability with increasing pressure (decreasing edge extension) and explain the non-monotonic behavior of the single knot formation probability, suggesting that our model provides an explanation of our experimental findings. Figure 5 in the manuscript compares the probability measurements obtained from the experimental data, the probability distribution predicted by the Poissonian model and our fitted model for different ranges of barrier extension R_{b}. At low pressures, the Poissonian model describes the experimental results very well. At relatively higher pressures (smaller R_{b}), on the other hand, Poissonian model fails to explain the experimentally obtained probabilities. We argue that the high compression limit where the Poisson model fails corresponds to knots forming in a limited range of positions close to the barrier at X=0. Simultaneous least-square fitting to the experimental one- and two-knot formation probabilities yields: $A_{\mathrm{b}} = 1.43 \pm 0.05$, $A_{\mathrm{wk}} = 0.98 \pm 0.12$ and $A_{\mathrm{h}} = 1.12 \pm 0.07$. The values of determined prefactors are on order of unity, required for self-consistency of the approach.

A feature of our free energy model (Supplementary Equation 33) is that it explicitly reduces to Dai *et al.*'s knotting free energy in the case where one knot is present and $V = 0$ (e.g. so that $R = 1$ everywhere along the chain). The most probable knot size is then determined by minimizing $f_1(L_{\mathrm{k}}, R = 1)$. We can explore if the values of A_{b} and A_{wk} obtained here can also describe Dai *et al.*'s simulation results for a chain in a no-flow equilibrium, a further test of self-consistency. Supplementary Figure **5** shows Dai *et al.*'s simulated most probable knot size versus channel width compared to the prediction of our free energy model (e.g. value of L_{k} that minimizes Supplementary Equation 33, with $R = 1$ and P set to the value used in Dai *et al.*'s simulations, $P = 0.4w$). We also show the model prediction using classic de Gennes bob statistics in Supplementary Equation 35 to estimate f_{wuk} (classic de Gennes theory, used by Dai *et al.*, is a more appropriate choice for channel widths exceeding around 400-500 nm, covering the range where the most probable knot sizes reach a maximum). The classic de Gennes prediction does not agree exactly, underestimating some of the points near the peak by around 10%, but it reasonably describes the overall trend in the

simulated results.

Alternatively, theory suggests fixing $A_{\mathrm{wk}} = 1$ and $A_{\mathrm{h}} = 1$ and then performing a *single parameter fit* to fix A_{b}. This approach gives agreement of equivalent quality with $A_{\mathrm{b}} = 1.46 \pm 0.01$ (see Supplementary Figure **6** for the comparison between the one parameter and three parameter fits). Note that the value of A_{b} is most critical to the overall agreement: too low and the knotting probability is too high for all R_{c}, too high and the knotting probability is suppressed relative to experiment. We believe that the slightly higher value of A_{b} relative to unity is required because our model may underestimate the knot confinement free energy. For example, slight compression of knots at high forcing, an effect not taken into account in our model, would lead to additional positive free energy contributions. The value of A_{b} might need to be increased upon fitting to compensate for the absence of these effects. We feel that these effects would be worthwhile to explore in future theoretical studies of knot-formation on compressed chains.

Note also that the predicted knot sizes are consistent with our single-file ordering hypothesis: two-knots cannot pass in the channel when the ratio of the knot diameter to largest channel dimension $2g_{\mathrm{k}}/D_2 > 0.5$. For the most probable knot size in a one-knot state, our theory predicts $2g_{\mathrm{k}}/D_2 > 0.6$ over the entire range of R_{b} values (degrees of compressed) used, so our theory self-consistently requires that single-file ordering occurs for multiple knot states.

Our statistical mechanical model also gives predictions for the spatial distributions of knots in steady-state (prior to pressure release). However, caution must be used in comparing our experimental results after pressure release with the predicted distribution in the compressed steady-state. Once the pressure is released, the hydrodynamic flow is no longer present, reducing the slope β of the free energy profile. In addition, during relaxation the concentration profile will flatten out with the profile becoming perfectly uniform in the long-time limit when the no-flow equilibrium is reached. These effects will lead to a dynamic variation in the free energy landscape felt by the knots which in turn will lead to a dynamic variation of the knot distribution. However, while these considerations mean we can't use the distributions following pressure release to draw strict quantitative conclusions about the knot distributions in steady-state, we can draw some qualitative conclusions. The one-knot distribution following pressure release has an exponential character (see Supplementary Figure **3**(a,c)) with the one-knot probability highest at the slit-barrier. We can infer

in steady-state that the knot distributions are also non-uniform with the knots accumulating at the slit-barrier ($x = 0$). Note that the relaxation process must increase entropy and therefore cannot introduce spatial non-uniformity into a profile that was initially uniform (i.e. it is not possible that the non-uniformity we observe was introduced by the relaxation process itself). In particular, we can say that the distributions observed after pressure release represent a lower-limit on the degree of spatial non-uniformity present in steady-state.

The lower knot distribution predicted by our theory, P_{21}, is given by Supplementary Equation 47; the upper knot distribution predicted, P_{22}, is given by Supplementary Equation 49 (see Supplementary Figure **7**(b)). If the two knots experience the same free energy profile with identical slope β, note that P_{L} and P_{21} have the same forms (compare Supplementary Equation 31 and Supplementary Equation 47), but that P_{U} and P_{22} are different. In particular, Supplementary Equation 49 predicts that P_{22} will be shifted to higher X relative to P_{U} (see Supplementary Figure **7**(c, d)), with the degree of shifting determined by $2g_{\mathrm{k}}$. In the limit g_{k} goes to zero, P_{U} and P_{22} coincide. Thus, we expect that repulsive interactions strong enough to prevent knot crossing will shift the upper knot distribution to higher X relative to P_{U}, inducing a spatial *segregation* between the distributions. Naturally, while we do not expect quantitative agreement between the steady-state distributions and our measured distributions (due to the complicated dynamics of the relaxation process), any shift observed between the lower and upper knot distributions is indicative of a no-passing condition. While the experimental spatial distribution in fact results from averaging over the distribution of all thermally allowed knot sizes, we find that averaging over all knot sizes has only a moderate effect on the predicted distributions and in fact would shift the upper knot distribution to higher X (see Supplementary Figure **7**(a, b)).

Supplementary Note 12

Model Predictions for Varying Channel Diameter

Our model gives predictions for how varying channel dimension should alter knotting probability in the extended de Gennes regime. We find that increasing confinement is predicted to increase the probability of knot generation (see Supplementary Figure **8**(a)). This is unsurprising given that increased confinement, by increasing the f_{wuk} term in the free energy will tend to enhance the free energy saved upon forming a knot, leading to a decreased single-knot free energy (Supplementary Figure **8**(b), red-curve). Our model also predicts that more multiple knot states would be generated for lower channel width (Supplementary Figure **8**(a)), due to the decreasing knot interaction free energy (Supplementary Figure **8**(b), blue-curve), which falls for channels below 500 nm due to the decreasing knot size with lower channel dimension (see Supplementary Figure **5**, the channel dimension necessarily places an upper limit on the knot size in our model). It would also be interesting to perform analogous experiments in the transition (< 100 nm) and sub-persistence Odijk regimes (< 50 nm). We do know that knots can be formed in channels on order of the persistence length [19], but our model will break down here as we do not expect the free energy scalings to extend to such small channels.

Supplementary Note 13

Experimental Error on Knotting Probability

Each knotting probability measurement (i.e. each data point in Fig. 3(b) and Fig. 4 in the manuscript) was obtained from around $10-15$ individual single-molecule compression-relaxation events. The compression ratio and waiting time shown were determined from averaging over all single molecule events corresponding to a given data point. The horizontal error bars give the corresponding error on the mean over the single-molecule data.

In the current study, we observe three possible outcomes: formation of 0, 1 and 2 knots ($i = 0, 1, 2$). The maximum likelihood estimate for the probability of a knotting state is $\hat{P}_i = n_i/N$ where n_i is the number of molecules in the given knotting state and N is the total number of events corresponding to the data point in question. The maximum likelihood estimate of total knotting probability is $\hat{P}_{\text{total}} = (n_1 + n_2)/N$. We report the maximum likelihood estimate of knotting probability in Figure 3(b) and Figure 4 in the manuscript. Our vertical error-bars are defined so that the true probability value lies within the bar with confidence $1 - \alpha$ with $\alpha = 0.32$ (for a standard one-sigma error). We argue that binomial statistics are appropriate for our measurements: we either find that the measurement falls within the specific category or outside the category (single knot present or not, two knots present or not, knots present or absent). We then estimate the confidence interval using the Wilson score interval [2]. Let $z^2 \equiv z^2_{\alpha/2}$, where $z_{\alpha/2}$ is the critical value of the normal distribution for error level α. The true knotting probability P_i then lies within the following interval with confidence $1 - \alpha$:

$$P_i \in (P_i' - \sigma_{\text{n}}, P_i' + \sigma_{\text{n}}) \tag{67}$$

where

$$\hat{P}_i' \equiv \frac{z^2 + 2n_i}{2(N + z^2)} \tag{68}$$

$$\sigma_{\text{n}} \equiv \frac{z}{2(N + z^2)} \left(\frac{z^2 + 4n_i(N - n_i)}{N} \right)^{1/2} \tag{69}$$

For a one-sigma error, $z_{\alpha/2} = 1$. Note that the confidence intervals are not distributed symmetrically around the maximum likelihood estimate $\hat{P}_i$. We thus use asymmetric error

bars to report error on knotting probability, with the top and bottom error bars defined via $\sigma_\mathrm{t} = \hat{P}_i' + \sigma_\mathrm{n} - \hat{P}_i$ and $\sigma_\mathrm{b} = \hat{P}_i - (\hat{P}_i' - \sigma_\mathrm{n})$, respectively.

Supplementary References

[1] Liang Dai, C Benjamin Renner, and Patrick S Doyle. Metastable knots in confined semiflexible chains. *Macromolecules*, 48(8):2812–2818, 2015.

[2] Robert G Newcombe. *Confidence intervals for proportions and related measures of effect size.* CRC Press, 2012.

[3] Walter Reisner, Jonas N Pedersen, and Robert H Austin. Dna confinement in nanochannels: physics and biological applications. *Reports on Progress in Physics*, 75(10):106601, 2012.

[4] Christoph G Baumann, Steven B Smith, Victor A Bloomfield, and Carlos Bustamante. Ionic effects on the elasticity of single dna molecules. *Proceedings of the National Academy of Sciences*, 94(12):6185–6190, 1997.

[5] Alexander R Klotz, Lyndon Duong, Mikhail Mamaev, Hendrick W de Haan, Jeff ZY Chen, and Walter W Reisner. Measuring the confinement free energy and effective width of single polymer chains via single-molecule tetris. *Macromolecules*, 48(14):5028–5033, 2015.

[6] Dirk Stigter. Interactions of highly charged colloidal cylinders with applications to double-stranded dna. *Biopolymers*, 16(7):1435–1448, 1977.

[7] Valentin V Rybenkov, Nicholas R Cozzarelli, and Alexander V Vologodskii. Probability of dna knotting and the effective diameter of the dna double helix. *Proceedings of the National Academy of Sciences*, 90(11):5307–5311, 1993.

[8] Ahmed Khorshid, Philip Zimny, David Tetreault-La Roche, Geremia Massarelli, Takahiro Sakaue, and Walter Reisner. Dynamic compression of single nanochannel confined dna via a nanodozer assay. *Phys. Rev. Lett.*, 113:268104, 2014.

[9] Ahmed Khorshid, Susan Amin, Zhiyue Zhang, Takahiro Sakaue, and Walter Reisner. Non-equilibrium dynamics of nanochannel confined dna. *Macromolecules*, 49(5):1933–1940, 2016.

[10] Frank H. J. van der Heyden, Derek Stein, and Cees Dekker. Streaming currents in a single nanofluidic channel. *Physical Review Letters*, 95:116104, 2005.

[11] Frank H. J. van der Heyden, Douwe Jan Bonthuis, Derek Stein, Christine Meyer, and Cees Dekker. Power generation by pressure-driven transport of ions in nanofluidic channels. *Nano Letters*, 7:1022–1025, 2007.

[12] Henrik Bruus. *Theoretical Microfluidics.* Oxford, 2007.

[13] Derek Stein, H. J. van der Heyden, Wiepke J. A. Koopmas, and C. Dekker. Pressure-driven

transport of confined dna polymers in fluidic channels. *Proced. Nat. Acad. Sci. USA*, 3:15853–15858, 2006.

[14] Alexander Grosberg and Yitzhak Rabin. Metastable tight knots in a wormlike polymer. *Phys. Rev. Lett.*, 99:217801, 2007.

[15] Liang Dai, Benjamin Renner, C, and Patrick Doyle. Metastable tight knots in semiflexible chains. *Macromolecules*, 47:6135–6140, 2014.

[16] Suckjoon Jun, Devarajan Thirumalai, and Bae-Yeun Ha. Compression and stretching of a self-avoiding chain in cylindrical nanopores. *Physical review letters*, 101(13):138101, 2008.

[17] Benjamin Trefz, Jonathan Siebert, and Peter Virnau. How molecular knots can pass through each other. *PNAS*, 111(22):7948–7951, 2014.

[18] H. D. Vollmer. Two particle model for the diffusion of interacting particles in periodic potentials. *Z. Physik B*, 33:103–109, 1979.

[19] Ralf Metzler, Walter Reisner, Robert Riehn, Robert Austin, JO Tegenfeldt, and Igor M Sokolov. Diffusion mechanisms of localised knots along a polymer. *EPL (Europhysics Letters)*, 76(4):696, 2006.
